# Supplementary material for: Molecular Characterization of Small Ruminant Lentiviruses in Sheep and Goats: A Systematic Review
Source: Animals (Basel). 2024 Dec 8;14(23):3545. doi: 10.3390/ani14233545 (PMC11640545; doi:10.3390/ani14233545)
Supplement: Supplementary file 1 [file animals-14-03545-s001.zip › Table S1.pdf]

Search strategy

	**PubMed: original search on 25.01.2023, updated on 25.01.2024**	**Search**	**Results**
#1	maedi OR maedi-visna OR maedi/visna OR caev OR lentivirus OR lentiviruses OR small ruminant lentiviruses OR srlv	Original search	130,599
		Updated search	133,630
#2	sheep OR goat OR goats OR ovine OR caprine OR small ruminant OR small ruminants	Original search	213,257
		Updated search	218,187
#3	phylogenetic analysis OR phylogenesis OR genetic analysis OR characterization OR genotyping OR classification OR subtype	Original search	4,039,662
		Updated search	4,257,069
#4	#1 AND #2 AND #3	Original search	460
		Updated search	467
#5	#1 AND #2 AND #3 Filters: English	Original search	450
		Updated search	457

	**Embase: original search on 25.01.2023, updated on 25.01.2024**	**Search**	**Results**
#1	maedi OR 'maedi-visna' OR 'maedi/visna' OR caev OR lentivirus OR lentiviruses OR 'small ruminant lentiviruses' OR srlv	Original search	263,933
		Updated search	275,024
#2	sheep OR goat OR goats OR ovine OR caprine OR 'small ruminant' OR 'small ruminants'	Original search	203,293
		Updated search	209,202
#3	'phylogenetic analysis' OR 'phylogeny' OR phylogenesis OR 'genetic analysis' OR characterization OR genotyping OR classification OR subtype	Original search	2,252,833
		Updated search	2,393,445
#4	#1 AND #2 AND #3	Original search	257
		Updated search	266
#5	#1 AND #2 AND #3 AND [english]/lim	Original search	249
		Updated search	258

	**Web of Science: original search on 25.01.2023, updated on 25.01.2024**	**Search**	**Results**
#1	TS=(maedi OR maedi-visna OR maedi/visna OR caev OR lentivirus OR lentiviruses OR small ruminant lentiviruses OR srlv)	Original search	15,488
		Updated search	16,523
#2	TS=(sheep OR goat OR goats OR ovine OR caprine OR small ruminant OR small ruminants)	Original search	196,899
		Updated search	203,803
#3	TS=(phylogenetic analysis OR phylogenesis OR genetic analysis OR characterization OR genotyping OR classification OR subtype)	Original search	3,856,492
		Updated search	4,116,402
#4	#1 AND #2 AND #3	Original search	287
		Updated search	300
#5	#1 AND #2 AND #3 and English (Languages)	Original search	276
		Updated search	289

	**Scopus: original search on 25.01.2023, updated on 25.01.2024**	**Search**	**Results**
#1	(maedi OR maedi-visna OR maedi/visna OR caev OR lentivirus OR lentiviruses OR small AND ruminant AND	Original search	296

	lentiviruses OR srlv) AND (sheep OR goat OR goats OR ovine OR caprine OR small AND ruminant OR small AND ruminants) AND (phylogenetic AND analysis OR phylogenesis OR genetic AND analysis OR characterization OR genotyping OR classification OR subtype) AND (LIMIT-TO (LANGUAGE , "English"))	Updated search	321
