# Supplementary material for: Molecular Characterization of Small Ruminant Lentiviruses in Sheep and Goats: A Systematic Review
Source: Animals (Basel). 2024 Dec 8;14(23):3545. doi: 10.3390/ani14233545 (PMC11640545; doi:10.3390/ani14233545)
Supplement: Supplementary file 1 [file animals-14-03545-s001.zip › Table S2.pdf]

Supplementary file 2. Full-text articles excluded and reasons for exclusion.

	#article	References	Reasons for exclusion
1	27	Angelopoulou, K., et al. (2006). "A novel deletion in the LTR region of a Greek small ruminant lentivirus may be associated with low pathogenicity." Virus Res 118(1-2): 178-184.	The study is not based on phylogenetic characterization, but on pathogenicity.
2	110	Campbell, B. J., et al. (1993). "Characterization of a New York ovine lentivirus isolate." J Gen Virol 74 (Pt 2): 201-210.	The study is based on sequences alignment, not on the analysis of phylogenetic trees and classification into genotypes and/or subgenotypes.
3	119	Caroline, L., et al. (2010). "SRLVs: A Genetic Continuum of Lentiviral Species in Sheep and Goats with Cumulative Evidence of Cross Species Transmission." Curr HIV Res 8(1): 94-100.	The study is a review based on the comparison between the pathogenicity of SRLVs and other Lentiviruses.
4	135	Celer Jr, V., et al. (2000). "The detection of proviral DNA by semi-nested polymerase chain reaction and phylogenetic analysis of Czech Maedi-Visna isolates based on gag gene sequences." Journal of Veterinary Medicine, Series B 47(3): 203-215.	The study is based on sequences alignment, not on the analysis of phylogenetic trees and classification into genotypes and/or subgenotypes.
5	136	Celer Jr, V., et al. (1997). "Isolation and partial characterization of ovine lentivirus in Czech Republic." Folia Microbiol (Praha) 42(4): 395-399.	The study is a genetic characterization based on electrophoresis gel fragments analysis.
6	278	Gazit, A., et al. (1983). "The caprine arthritis-encephalitis virus is a distinct virus within the lentivirus group." Virology 124(1): 192-195.	The study is a genetic characterization based on hybridization assays.
7	383	Irving, S. G., et al. (1984). "Isolation and characterization of a novel retrovirus from sheep affected by pulmonary carcinoma." Virology 134(1): 244-248.	The study is a genetic characterization based on hybridization assays.
8	403	Kaba, J., et al. (2009). "Isolation and characterization of caprine arthritis encephalitis virus in goats from Poland." Pol J Vet Sci 12(2): 183-188.	The study is based on sequences alignment, not on the analysis of phylogenetic trees and classification into genotypes and/or subgenotypes.
9	462	Leroux, C., et al. (1996). "Molecular characterization of field isolates of lentiviruses of small ruminants." AIDS Res Hum Retroviruses 12(5): 427-429.	The study is based on a comparison between SRLVs and HIV.
10	463	Leroux, C., et al. (1995). "Genomic	The study is based on a comparison between

		heterogeneity in the pol region of ovine lentiviruses obtained from bronchoalveolar cells of infected sheep from France." J Gen Virol 76 (Pt 6): 1533-1537.	SRLVs and HIV.
11	463	Letko, A., et al. (2021). "Genetic evaluation of small ruminant lentivirus susceptibility in Valais blacknose sheep." Anim Genet 52(5): 781-782.	The study is an evaluation of the SRLVs susceptibility in Valais blacknose sheep.
12	547	Murphy, B., et al. (2010). "Tissue tropism and promoter sequence variation in caprine arthritis encephalitis virus infected goats." Virus Res 151(2): 177-184.	Study based on tissue tropism and promoter sequence variation analysis.
13	569	Olech, M., et al. (2009). "EVIDENCE FOR INTERSPECIES TRANSMISSION OF SMALL RUMINANT LENTIVIRUSES IN SHEEP AND GOATS IN POLAND." BULLETIN OF THE VETERINARY INSTITUTE IN PULAWY 53(2): 165-168.	Study based on heteroduplex mobility assay.
14	588	Olech, M., et al. (2012). "Diversity of gag gene sequence encoding immunodominant epitope on capsid protein of lentiviruses from sheep in Poland." BULLETIN OF THE VETERINARY INSTITUTE IN PULAWY 56(4): 431-434.	The study is based on sequences alignment, not on the analysis of phylogenetic trees and classification into genotypes and/or subgenotypes.
15	653	Querat, G., et al. (1990). "Nucleotide sequence analysis of SA-OMVV, a visna-related ovine lentivirus: phylogenetic history of lentiviruses." Virology 175(2): 434-447.	The study is based on sequences alignment, not on the analysis of phylogenetic trees and classification into genotypes and/or subgenotypes.
16	665	Ramirez, H., et al. (2012). "Study of compartmentalization in the visna clinical form of small ruminant lentivirus infection in sheep." BMC Vet Res 8.	The study is based on SRLVs compartmentalization, not on the analysis of phylogenetic trees and classification into genotypes and/or subgenotypes.
17	686	Rosati, S., et al. (1995). "Genome Analysis of North American Small Ruminant Lentiviruses by Polymerase Chain Reaction and Restriction Enzyme Analysis." Journal of Veterinary Diagnostic Investigation 7(4): 437-443.	The study is based on PCR and restriction enzyme analysis.
18	690	Rosati, S., et al. (2004). "Antigenic variability of ovine lentivirus isolated in Italy." Vet Res Commun 28 Suppl 1: 319-322.	Study based on previously obtained sequences and focused more on characterisation of antigenic variability.

19	707	Sargan, D. R., et al. (1991). "Nucleotide sequence of EV1, a British isolate of maedi-visna virus." J Gen Virol 72 (Pt 8): 1893-1903.	The study is based on sequences alignment, not on the analysis of phylogenetic trees and classification into genotypes and/or subgenotypes.
20	724	Sherman, L., et al. (1986). "Nucleotide sequence analysis of the long terminal repeat of integrated caprine arthritis encephalitis virus." Virus Res 5(2-3): 145-155.	The study is based on sequences alignment, not on the analysis of phylogenetic trees and classification into genotypes and/or subgenotypes.
21	770	Tonka, T., et al. (2019). "The phylogenetic analysis of a novel genetic subtype of caprine arthritis encephalitis virus (CAEV) in the Czech Republic." Acta Virol 63(2): 240-242.	Letter to the editor.
22	827	Zanoni, R. G., et al. (1992). "Genomic heterogeneity of small ruminant lentiviruses detected by PCR." Vet Microbiol 33(1-4): 341-351.	The study is based on PCR and restriction enzyme analysis.
23	828	Zanoni, R. G., et al. (1991). "Expression in Escherichia coli and sequencing of the coding region for the capsid protein of Dutch maedi-visna virus strain ZZV 1050: application of recombinant protein in enzyme-linked immunosorbent assay for the detection of caprine and ovine lentiviruses." J Clin Microbiol 29(7): 1290-1294.	The study is based on the synthesis and analysis of a recombinant gag fusion protein.
