# Supplementary material for: Molecular Characterization of Small Ruminant Lentiviruses in Sheep and Goats: A Systematic Review
Source: Animals (Basel). 2024 Dec 8;14(23):3545. doi: 10.3390/ani14233545 (PMC11640545; doi:10.3390/ani14233545)
Supplement: Supplementary file 1 [file animals-14-03545-s001.zip › Table S3.pdf]

JBI CRITICAL APPRAISAL CHECKLIST FOR ANALYTICAL CROSS SECTIONAL STUDIES

Reviewer SILVIA PAVONE/PAOLA GOBBI Date________10/08/2023_____________

Author Acevedo Jimenez et al. Year 2021 Record Number 1

	Yes	No	Unclear	Not applicable
1. Were the criteria for inclusion in the sample clearly defined?	■	□	□	□
2. Were the study subjects and the setting described in detail?	□	■	□	□
3. Was the exposure measured in a valid and reliable way?	□	■	□	□
4. Were objective, standard criteria used for measurement of the condition?	■	□	□	□
5. Were confounding factors identified?	□	□	□	■
6. Were strategies to deal with confounding factors stated?	□	□	□	■
7. Were the outcomes measured in a valid and reliable way and expressed clearly? Has been used any reliable statistical methods to evaluate the phylogenetic results?	□	■	□	□
8. Was appropriate statistical analysis used?	■	□	□	□

Overall appraisal: Include ■ Exclude □ Seek further info □

Comments (Including reason for exclusion):

Criticality: missing data (time period), there is no correlation between the accession numbers used and the subgenotypes, the original identifiers are given in the tree, unsatisfactory medical history and signalment

Ranking: unsatisfactory (50%)

Reviewer SILVIA PAVONE/PAOLA GOBBI Date_________10/08/2023______________

Author Angelopoulou et al., Year 2005 Record Number 3

	Yes	No	Unclear	Not applicable
1. Were the criteria for inclusion in the sample clearly defined?	■	☐	☐	☐
2. Were the study subjects and the setting described in detail?	☐	■	☐	☐
3. Was the exposure measured in a valid and reliable way?	■	☐	☐	☐
4. Were objective, standard criteria used for measurement of the condition?	■	☐	☐	☐
5. Were confounding factors identified?	☐	☐	☐	■
6. Were strategies to deal with confounding factors stated?	☐	☐	☐	■
7. Were the outcomes measured in a valid and reliable way and expressed clearly? Has been used any reliable statistical methods to evaluate the phylogenetic results?	☐	■	☐	☐
8. Was appropriate statistical analysis used?	■	☐	☐	☐

Overall appraisal: Include ■ Exclude ☐ Seek further info ☐

Comments (Including reason for exclusion)

Criticality: missing data (time period, subgenotypes)

Ranking: good (66%)

© JBI, 2020. All rights reserved. JBI grants use of these tools for research purposes only. All other enquiries should be sent to jbisynthesis@adelaide.edu.au.

Reviewer SILVIA PAVONE/PAOLA GOBBI Date_________10/08/2023______________

Author Arcangeli et al., Year 2022 Record Number 4

	Yes	No	Unclear	Not applicable
1. Were the criteria for inclusion in the sample clearly defined?	■	□	□	□
2. Were the study subjects and the setting described in detail?	■	□	□	□
3. Was the exposure measured in a valid and reliable way?	■	□	□	□
4. Were objective, standard criteria used for measurement of the condition?	■	□	□	□
5. Were confounding factors identified?	□	□	□	■
6. Were strategies to deal with confounding factors stated?	□	□	□	■
7. Were the outcomes measured in a valid and reliable way and expressed clearly? Has been used any reliable statistical methods to evaluate the phylogenetic results?	■	□	□	□
8. Was appropriate statistical analysis used?	■	□	□	□

Overall appraisal: Include ■ Exclude □ Seek further info □

Comments (Including reason for exclusion)

Ranking: very good (100%)

© JBI, 2020. All rights reserved. JBI grants use of these tools for research purposes only. All other enquiries should be sent to jbisynthesis@adelaide.edu.au.

Reviewer SILVIA PAVONE/PAOLA GOBBI Date__________10/08/2023________________

Author Barros et al., Year 2004 Record Number 5

	Yes	No	Unclear	Not applicable
1. Were the criteria for inclusion in the sample clearly defined?	■	□	□	□
2. Were the study subjects and the setting described in detail?	□	■	□	□
3. Was the exposure measured in a valid and reliable way?	■	□	□	□
4. Were objective, standard criteria used for measurement of the condition?	■	□	□	□
5. Were confounding factors identified?	□	□	□	■
6. Were strategies to deal with confounding factors stated?	□	□	□	■
7. Were the outcomes measured in a valid and reliable way and expressed clearly? Has been used any reliable statistical methods to evaluate the phylogenetic results?	■	□	□	□
8. Was appropriate statistical analysis used?	■	□	□	□

Overall appraisal: Include ■ Exclude □ Seek further info □

Comments (Including reason for exclusion)

Criticality: missing data (time period)

Ranking: very good (83%)

© JBI, 2020. All rights reserved. JBI grants use of these tools for research purposes only. All other enquiries should be sent to jbisynthesis@adelaide.edu.au.

Reviewer SILVIA PAVONE/PAOLA GOBBI Date__________10/08/2023_______________

Author Bartak et al., Year 2018 Record Number 6

	Yes	No	Unclear	Not applicable
1. Were the criteria for inclusion in the sample clearly defined?	■	□	□	□
2. Were the study subjects and the setting described in detail?	■	□	□	□
3. Was the exposure measured in a valid and reliable way?	■	□	□	□
4. Were objective, standard criteria used for measurement of the condition?	■	□	□	□
5. Were confounding factors identified?	□	□	□	■
6. Were strategies to deal with confounding factors stated?	□	□	□	■
7. Were the outcomes measured in a valid and reliable way and expressed clearly? Has been used any reliable statistical methods to evaluate the phylogenetic results?	□	■	□	□
8. Was appropriate statistical analysis used?	■	□	□	□

Overall appraisal: Include ■ Exclude □ Seek further info □

Comments (Including reason for exclusion)

Criticality: missing data (accession numbers), authors claim to have found A2 and A3 subgenotypes and B genotype but they do not correlate specifically these data to the samples

Ranking: very good (100%)

© JBI, 2020. All rights reserved. JBI grants use of these tools for research purposes only. All other enquiries should be sent to jbisynthesis@adelaide.edu.au.

Reviewer SILVIA PAVONE/PAOLA GOBBI Date_________10/08/2023______________

Author Bazzucchi et al., Year 2021 Record Number 7

	Yes	No	Unclear	Not applicable
1. Were the criteria for inclusion in the sample clearly defined?	■	□	□	□
2. Were the study subjects and the setting described in detail?	■	□	□	□
3. Was the exposure measured in a valid and reliable way?	■	□	□	□
4. Were objective, standard criteria used for measurement of the condition?	■	□	□	□
5. Were confounding factors identified?	□	□	□	■
6. Were strategies to deal with confounding factors stated?	□	□	□	■
7. Were the outcomes measured in a valid and reliable way and expressed clearly? Has been used any reliable statistical methods to evaluate the phylogenetic results?	■	□	□	□
8. Was appropriate statistical analysis used?	■	□	□	□

Overall appraisal: Include ■ Exclude □ Seek further info □

Comments (Including reason for exclusion)

Ranking: very good (100%)

© JBI, 2020. All rights reserved. JBI grants use of these tools for research purposes only. All other enquiries should be sent to jbisynthesis@adelaide.edu.au.

Reviewer SILVIA PAVONE/PAOLA GOBBI Date_________10/08/2023______________

Author Bertolotti et al., Year 2011 Record Number 8

	Yes	No	Unclear	Not applicable
1. Were the criteria for inclusion in the sample clearly defined?	■	□	□	□
2. Were the study subjects and the setting described in detail?	□	■	□	□
3. Was the exposure measured in a valid and reliable way?	■	□	□	□
4. Were objective, standard criteria used for measurement of the condition?	■	□	□	□
5. Were confounding factors identified?	□	□	□	■
6. Were strategies to deal with confounding factors stated?	□	□	□	■
7. Were the outcomes measured in a valid and reliable way and expressed clearly? Has been used any reliable statistical methods to evaluate the phylogenetic results?	■	□	□	□
8. Was appropriate statistical analysis used?	■	□	□	□

Overall appraisal: Include ■ Exclude □ Seek further info □

Comments (Including reason for exclusion)

Criticality: missing data (time period)

Ranking: very good (83%)

© JBI, 2020. All rights reserved. JBI grants use of these tools for research purposes only. All other enquiries should be sent to jbisynthesis@adelaide.edu.au.

Reviewer SILVIA PAVONE/PAOLA GOBBI Date__________10/08/2023_______________

Author Blatti-Cardinaux et al., Year 2016 Record Number 9

	Yes	No	Unclear	Not applicable
1. Were the criteria for inclusion in the sample clearly defined?	■	□	□	□
2. Were the study subjects and the setting described in detail?	□	■	□	□
3. Was the exposure measured in a valid and reliable way?	■	□	□	□
4. Were objective, standard criteria used for measurement of the condition?	■	□	□	□
5. Were confounding factors identified?	□	□	□	■
6. Were strategies to deal with confounding factors stated?	□	□	□	■
7. Were the outcomes measured in a valid and reliable way and expressed clearly? Has been used any reliable statistical methods to evaluate the phylogenetic results?	■	□	□	□
8. Was appropriate statistical analysis used?	■	□	□	□

Overall appraisal: Include ■ Exclude □ Seek further info □

Comments (Including reason for exclusion)

Criticality: missing data (time period)

Ranking: very good (83%)

© JBI, 2020. All rights reserved. JBI grants use of these tools for research purposes only. All other enquiries should be sent to jbisynthesis@adelaide.edu.au.

Reviewer SILVIA PAVONE/PAOLA GOBBI Date_________10/08/2023______________

Author Braz et al., Year 2022 Record Number 10

	Yes	No	Unclear	Not applicable
1. Were the criteria for inclusion in the sample clearly defined?	■	□	□	□
2. Were the study subjects and the setting described in detail?	■	□	□	□
3. Was the exposure measured in a valid and reliable way?	■	□	□	□
4. Were objective, standard criteria used for measurement of the condition?	■	□	□	□
5. Were confounding factors identified?	□	□	□	■
6. Were strategies to deal with confounding factors stated?	□	□	□	■
7. Were the outcomes measured in a valid and reliable way and expressed clearly? Has been used any reliable statistical methods to evaluate the phylogenetic results?	□	■	□	□
8. Was appropriate statistical analysis used?	■	□	□	□

Overall appraisal: Include ■ Exclude □ Seek further info □

Comments (Including reason for exclusion)

Criticality: low bootstrap values in phylogenetic trees

Ranking: very good (83%)

© JBI, 2020. All rights reserved. JBI grants use of these tools for research purposes only. All other enquiries should be sent to jbisynthesis@adelaide.edu.au.

Reviewer SILVIA PAVONE/PAOLA GOBBI Date__________10/08/2023_______________

Author Cardinaux et al., Year 2013 Record Number 11

	Yes	No	Unclear	Not applicable
1. Were the criteria for inclusion in the sample clearly defined?	■	□	□	□
2. Were the study subjects and the setting described in detail?	□	■	□	□
3. Was the exposure measured in a valid and reliable way?	□	■	□	□
4. Were objective, standard criteria used for measurement of the condition?	■	□	□	□
5. Were confounding factors identified?	□	□	□	■
6. Were strategies to deal with confounding factors stated?	□	□	□	■
7. Were the outcomes measured in a valid and reliable way and expressed clearly? Has been used any reliable statistical methods to evaluate the phylogenetic results?	□	■	□	□
8. Was appropriate statistical analysis used?	■	□	□	□

Overall appraisal: Include ■ Exclude □ Seek further info □

Comments (Including reason for exclusion)

Criticality: missing data (time period, accession numbers), low bootstrap values in phylogenetic trees

Ranking: unsatisfactory (50%)

© JBI, 2020. All rights reserved. JBI grants use of these tools for research purposes only. All other enquiries should be sent to jbisynthesis@adelaide.edu.au.

Reviewer SILVIA PAVONE/PAOLA GOBBI Date_________10/08/2023______________

Author Castro et al., Year 1999 Record Number 12

	Yes	No	Unclear	Not applicable
1. Were the criteria for inclusion in the sample clearly defined?	■	□	□	□
2. Were the study subjects and the setting described in detail?	□	■	□	□
3. Was the exposure measured in a valid and reliable way?	■	□	□	□
4. Were objective, standard criteria used for measurement of the condition?	■	□	□	□
5. Were confounding factors identified?	□	□	□	■
6. Were strategies to deal with confounding factors stated?	□	□	□	■
7. Were the outcomes measured in a valid and reliable way and expressed clearly? Has been used any reliable statistical methods to evaluate the phylogenetic results?	□	■	□	□
8. Was appropriate statistical analysis used?	■	□	□	□

Overall appraisal: Include ■ Exclude □ Seek further info □

Comments (Including reason for exclusion)

Criticality: missing data (time period, accession numbers, subgenotypes)

Ranking: good (66%)

© JBI, 2020. All rights reserved. JBI grants use of these tools for research purposes only. All other enquiries should be sent to jbisynthesis@adelaide.edu.au.

Reviewer SILVIA PAVONE/PAOLA GOBBI Date_________10/08/2023______________

Author Chassalevris et al., Year 2020 Record Number 14

	Yes	No	Unclear	Not applicable
1. Were the criteria for inclusion in the sample clearly defined?	■	□	□	□
2. Were the study subjects and the setting described in detail?	□	■	□	□
3. Was the exposure measured in a valid and reliable way?	■	□	□	□
4. Were objective, standard criteria used for measurement of the condition?	■	□	□	□
5. Were confounding factors identified?	□	□	□	■
6. Were strategies to deal with confounding factors stated?	□	□	□	■
7. Were the outcomes measured in a valid and reliable way and expressed clearly? Has been used any reliable statistical methods to evaluate the phylogenetic results?	■	□	□	□
8. Was appropriate statistical analysis used?	■	□	□	□

Overall appraisal: Include ■ Exclude □ Seek further info □

Comments (Including reason for exclusion)

Criticality: missing data (time period)

Ranking: very good (83%)

© JBI, 2020. All rights reserved. JBI grants use of these tools for research purposes only. All other enquiries should be sent to jbisynthesis@adelaide.edu.au.

Reviewer SILVIA PAVONE/PAOLA GOBBI Date__________10/08/2023_______________

Author Chebloune et al., Year 1996 Record Number 15

	Yes	No	Unclear	Not applicable
1. Were the criteria for inclusion in the sample clearly defined?	■	□	□	□
2. Were the study subjects and the setting described in detail?	□	■	□	□
3. Was the exposure measured in a valid and reliable way?	■	□	□	□
4. Were objective, standard criteria used for measurement of the condition?	■	□	□	□
5. Were confounding factors identified?	□	□	□	■
6. Were strategies to deal with confounding factors stated?	□	□	□	■
7. Were the outcomes measured in a valid and reliable way and expressed clearly? Has been used any reliable statistical methods to evaluate the phylogenetic results?	□	■	□	□
8. Was appropriate statistical analysis used?	□	■	□	□

Overall appraisal: Include ■ Exclude □ Seek further info □

Comments (Including reason for exclusion)

Criticality: missing data (time period, tree-building, bootstrap values)

Ranking: unsatisfactory (50%)

© JBI, 2020. All rights reserved. JBI grants use of these tools for research purposes only. All other enquiries should be sent to jbisynthesis@adelaide.edu.au.

Reviewer SILVIA PAVONE/PAOLA GOBBI Date_________10/08/2023______________

Author Clawson et al., Year 2015 Record Number 16

	Yes	No	Unclear	Not applicable
1. Were the criteria for inclusion in the sample clearly defined?	■	□	□	□
2. Were the study subjects and the setting described in detail?	□	■	□	□
3. Was the exposure measured in a valid and reliable way?	■	□	□	□
4. Were objective, standard criteria used for measurement of the condition?	■	□	□	□
5. Were confounding factors identified?	□	□	□	■
6. Were strategies to deal with confounding factors stated?	□	□	□	■
7. Were the outcomes measured in a valid and reliable way and expressed clearly? Has been used any reliable statistical methods to evaluate the phylogenetic results?	■	□	□	□
8. Was appropriate statistical analysis used?	■	□	□	□

Overall appraisal: Include ■ Exclude □ Seek further info □

Comments (Including reason for exclusion)

Criticality: missing data (time period)

Ranking: very good (83%)

© JBI, 2020. All rights reserved. JBI grants use of these tools for research purposes only. All other enquiries should be sent to jbisynthesis@adelaide.edu.au.

Reviewer SILVIA PAVONE/PAOLA GOBBI Date__________10/08/2023_______________

Author Colitti et al., Year 2019 Record Number 17

	Yes	No	Unclear	Not applicable
1. Were the criteria for inclusion in the sample clearly defined?	■	□	□	□
2. Were the study subjects and the setting described in detail?	□	■	□	□
3. Was the exposure measured in a valid and reliable way?	■	□	□	□
4. Were objective, standard criteria used for measurement of the condition?	■	□	□	□
5. Were confounding factors identified?	□	□	□	■
6. Were strategies to deal with confounding factors stated?	□	□	□	■
7. Were the outcomes measured in a valid and reliable way and expressed clearly? Has been used any reliable statistical methods to evaluate the phylogenetic results?	■	□	□	□
8. Was appropriate statistical analysis used?	■	□	□	□

Overall appraisal: Include ■ Exclude □ Seek further info □

Comments (Including reason for exclusion)

Criticality: missing data (time period)

Ranking: very good (83%)

© JBI, 2020. All rights reserved. JBI grants use of these tools for research purposes only. All other enquiries should be sent to jbisynthesis@adelaide.edu.au.

Reviewer SILVIA PAVONE/PAOLA GOBBI Date_________10/08/2023______________

Author Azevedo et al., Year 2019 Record Number 18

	Yes	No	Unclear	Not applicable
1. Were the criteria for inclusion in the sample clearly defined?	■	□	□	□
2. Were the study subjects and the setting described in detail?	□	■	□	□
3. Was the exposure measured in a valid and reliable way?	■	□	□	□
4. Were objective, standard criteria used for measurement of the condition?	■	□	□	□
5. Were confounding factors identified?	□	□	□	■
6. Were strategies to deal with confounding factors stated?	□	□	□	■
7. Were the outcomes measured in a valid and reliable way and expressed clearly? Has been used any reliable statistical methods to evaluate the phylogenetic results?	□	■	□	□
8. Was appropriate statistical analysis used?	■	□	□	□

Overall appraisal: Include ■ Exclude □ Seek further info □

Comments (Including reason for exclusion)

Criticality: missing data (time period), short genomic region used as target, low bootstrap values expressed in phylogenetic tree

Ranking: good (66%)

© JBI, 2020. All rights reserved. JBI grants use of these tools for research purposes only. All other enquiries should be sent to jbisynthesis@adelaide.edu.au.

Reviewer SILVIA PAVONE/PAOLA GOBBI Date__________10/08/2023________________

Author Luz-Armendariz et al., Year 2021 Record Number 19

	Yes	No	Unclear	Not applicable
1. Were the criteria for inclusion in the sample clearly defined?	■	□	□	□
2. Were the study subjects and the setting described in detail?	■	□	□	□
3. Was the exposure measured in a valid and reliable way?	■	□	□	□
4. Were objective, standard criteria used for measurement of the condition?	■	□	□	□
5. Were confounding factors identified?	□	□	□	■
6. Were strategies to deal with confounding factors stated?	□	□	□	■
7. Were the outcomes measured in a valid and reliable way and expressed clearly? Has been used any reliable statistical methods to evaluate the phylogenetic results?	□	■	□	□
8. Was appropriate statistical analysis used?	■	□	□	□

Overall appraisal: Include ■ Exclude □ Seek further info □

Comments (Including reason for exclusion)

Criticality: it is unclear how the authors obtained the 9131 bp sequence (contigs assembly?), low bootstrap values expressed in phylogenetic tree

Ranking: very good (83%)

© JBI, 2020. All rights reserved. JBI grants use of these tools for research purposes only. All other enquiries should be sent to jbisynthesis@adelaide.edu.au.

Reviewer SILVIA PAVONE/PAOLA GOBBI Date_________10/08/2023______________

Author De Martin et al., Year 2019 Record Number 20

	Yes	No	Unclear	Not applicable
1. Were the criteria for inclusion in the sample clearly defined?	■	□	□	□
2. Were the study subjects and the setting described in detail?	■	□	□	□
3. Was the exposure measured in a valid and reliable way?	■	□	□	□
4. Were objective, standard criteria used for measurement of the condition?	■	□	□	□
5. Were confounding factors identified?	□	□	□	■
6. Were strategies to deal with confounding factors stated?	□	□	□	■
7. Were the outcomes measured in a valid and reliable way and expressed clearly? Has been used any reliable statistical methods to evaluate the phylogenetic results?	□	■	□	□
8. Was appropriate statistical analysis used?	■	□	□	□

Overall appraisal: Include ■ Exclude □ Seek further info □

Comments (Including reason for exclusion)

Criticality: missing data (accession numbers, sequencing technique)

Ranking: very good (83%)

© JBI, 2020. All rights reserved. JBI grants use of these tools for research purposes only. All other enquiries should be sent to jbisynthesis@adelaide.edu.au.

Reviewer SILVIA PAVONE/PAOLA GOBBI Date_________10/08/2023______________

Author Deubelbeiss et al., Year 2014 Record Number 21

	Yes	No	Unclear	Not applicable
1. Were the criteria for inclusion in the sample clearly defined?	■	□	□	□
2. Were the study subjects and the setting described in detail?	□	■	□	□
3. Was the exposure measured in a valid and reliable way?	■	□	□	□
4. Were objective, standard criteria used for measurement of the condition?	■	□	□	□
5. Were confounding factors identified?	□	□	□	■
6. Were strategies to deal with confounding factors stated?	□	□	□	■
7. Were the outcomes measured in a valid and reliable way and expressed clearly? Has been used any reliable statistical methods to evaluate the phylogenetic results?	■	□	□	□
8. Was appropriate statistical analysis used?	■	□	□	□

Overall appraisal: Include ■ Exclude □ Seek further info □

Comments (Including reason for exclusion)

Criticality: missing data (time period)

Ranking: very good (83%)

© JBI, 2020. All rights reserved. JBI grants use of these tools for research purposes only. All other enquiries should be sent to jbisynthesis@adelaide.edu.au.

Reviewer SILVIA PAVONE/PAOLA GOBBI Date_________10/08/2023______________

Author Dickey et al., Year 2022 Record Number 22

	Yes	No	Unclear	Not applicable
1. Were the criteria for inclusion in the sample clearly defined?	■	□	□	□
2. Were the study subjects and the setting described in detail?	□	■	□	□
3. Was the exposure measured in a valid and reliable way?	■	□	□	□
4. Were objective, standard criteria used for measurement of the condition?	■	□	□	□
5. Were confounding factors identified?	□	□	□	■
6. Were strategies to deal with confounding factors stated?	□	□	□	■
7. Were the outcomes measured in a valid and reliable way and expressed clearly? Has been used any reliable statistical methods to evaluate the phylogenetic results?	■	□	□	□
8. Was appropriate statistical analysis used?	□	■	□	□

Overall appraisal: Include ■ Exclude □ Seek further info □

Comments (Including reason for exclusion)

Criticality: missing data (time period, bootstrap values)

Ranking: good (66%)

© JBI, 2020. All rights reserved. JBI grants use of these tools for research purposes only. All other enquiries should be sent to jbisynthesis@adelaide.edu.au.

Reviewer SILVIA PAVONE/PAOLA GOBBI Date__________10/08/2023_______________

Author Elfahal et al., Year 2010 Record Number 23

	Yes	No	Unclear	Not applicable
1. Were the criteria for inclusion in the sample clearly defined?	■	□	□	□
2. Were the study subjects and the setting described in detail?	□	■	□	□
3. Was the exposure measured in a valid and reliable way?	□	■	□	□
4. Were objective, standard criteria used for measurement of the condition?	■	□	□	□
5. Were confounding factors identified?	□	□	□	■
6. Were strategies to deal with confounding factors stated?	□	□	□	■
7. Were the outcomes measured in a valid and reliable way and expressed clearly? Has been used any reliable statistical methods to evaluate the phylogenetic results?	■	□	□	□
8. Was appropriate statistical analysis used?	□	■	□	□

Overall appraisal: Include ■ Exclude □ Seek further info □

Comments (Including reason for exclusion)

Criticality: missing data (time period, exposition, bootstrap values)

Ranking: unsatisfactory (50%)

© JBI, 2020. All rights reserved. JBI grants use of these tools for research purposes only. All other enquiries should be sent to jbisynthesis@adelaide.edu.au.

Reviewer SILVIA PAVONE/PAOLA GOBBI Date__________10/08/2023_______________

Author Feitosa et al., Year 2010 Record Number 24

	Yes	No	Unclear	Not applicable
1. Were the criteria for inclusion in the sample clearly defined?	■	☐	☐	☐
2. Were the study subjects and the setting described in detail?	☐	■	☐	☐
3. Was the exposure measured in a valid and reliable way?	■	☐	☐	☐
4. Were objective, standard criteria used for measurement of the condition?	■	☐	☐	☐
5. Were confounding factors identified?	☐	☐	☐	■
6. Were strategies to deal with confounding factors stated?	☐	☐	☐	■
7. Were the outcomes measured in a valid and reliable way and expressed clearly? Has been used any reliable statistical methods to evaluate the phylogenetic results?	☐	■	☐	☐
8. Was appropriate statistical analysis used?	■	☐	☐	☐

Overall appraisal: Include ■ Exclude ☐ Seek further info ☐

Comments (Including reason for exclusion)

Criticality: missing data (time period, sequencing technique), low bootstrap values expressed in phylogenetic tree

Ranking: good (66%)

© JBI, 2020. All rights reserved. JBI grants use of these tools for research purposes only. All other enquiries should be sent to jbisynthesis@adelaide.edu.au.

Reviewer SILVIA PAVONE/PAOLA GOBBI Date_________10/08/2023______________

Author Fras et al., Year 2013 Record Number 25

	Yes	No	Unclear	Not applicable
1. Were the criteria for inclusion in the sample clearly defined?	■	□	□	□
2. Were the study subjects and the setting described in detail?	□	■	□	□
3. Was the exposure measured in a valid and reliable way?	■	□	□	□
4. Were objective, standard criteria used for measurement of the condition?	■	□	□	□
5. Were confounding factors identified?	□	□	□	■
6. Were strategies to deal with confounding factors stated?	□	□	□	■
7. Were the outcomes measured in a valid and reliable way and expressed clearly? Has been used any reliable statistical methods to evaluate the phylogenetic results?	□	■	□	□
8. Was appropriate statistical analysis used?	■	□	□	□

Overall appraisal: Include ■ Exclude □ Seek further info □

Comments (Including reason for exclusion)

Criticality: missing data (time period), there is no correlation between accession numbers and subgenotypes

Ranking: good (66%)

© JBI, 2020. All rights reserved. JBI grants use of these tools for research purposes only. All other enquiries should be sent to jbisynthesis@adelaide.edu.au.

Reviewer SILVIA PAVONE/PAOLA GOBBI Date_________10/08/2023______________

Author Gayo et al., Year 2018 Record Number 26

	Yes	No	Unclear	Not applicable
1. Were the criteria for inclusion in the sample clearly defined?	■	□	□	□
2. Were the study subjects and the setting described in detail?	■	□	□	□
3. Was the exposure measured in a valid and reliable way?	■	□	□	□
4. Were objective, standard criteria used for measurement of the condition?	■	□	□	□
5. Were confounding factors identified?	□	□	□	■
6. Were strategies to deal with confounding factors stated?	□	□	□	■
7. Were the outcomes measured in a valid and reliable way and expressed clearly? Has been used any reliable statistical methods to evaluate the phylogenetic results?	□	■	□	□
8. Was appropriate statistical analysis used?	■	□	□	□

Overall appraisal: Include ■ Exclude □ Seek further info □

Comments (Including reason for exclusion)

Criticality: LTR phylogenetic tree based on short genomic region

Ranking: very good (83%)

© JBI, 2020. All rights reserved. JBI grants use of these tools for research purposes only. All other enquiries should be sent to jbisynthesis@adelaide.edu.au.

Reviewer SILVIA PAVONE/PAOLA GOBBI Date_________17/08/2023______________

Author Germain et al., Year 2008 Record Number 27

	Yes	No	Unclear	Not applicable
1. Were the criteria for inclusion in the sample clearly defined?	■	□	□	□
2. Were the study subjects and the setting described in detail?	□	■	□	□
3. Was the exposure measured in a valid and reliable way?	■	□	□	□
4. Were objective, standard criteria used for measurement of the condition?	■	□	□	□
5. Were confounding factors identified?	□	□	□	■
6. Were strategies to deal with confounding factors stated?	□	□	□	■
7. Were the outcomes measured in a valid and reliable way and expressed clearly? Has been used any reliable statistical methods to evaluate the phylogenetic results?	□	■	□	□
8. Was appropriate statistical analysis used?	■	□	□	□

Overall appraisal: Include ■ Exclude □ Seek further info □

Comments (Including reason for exclusion)

Criticality: missing data (time period), there is no correlation between accession numbers and subgenotypes

Ranking: good (66%)

© JBI, 2020. All rights reserved. JBI grants use of these tools for research purposes only. All other enquiries should be sent to jbisynthesis@adelaide.edu.au.

Reviewer SILVIA PAVONE/PAOLA GOBBI Date_________17/08/2023______________

Author Germain and Valas, Year 2006 Record Number 28

	Yes	No	Unclear	Not applicable
1. Were the criteria for inclusion in the sample clearly defined?	■	□	□	□
2. Were the study subjects and the setting described in detail?	□	■	□	□
3. Was the exposure measured in a valid and reliable way?	■	□	□	□
4. Were objective, standard criteria used for measurement of the condition?	■	□	□	□
5. Were confounding factors identified?	□	□	□	■
6. Were strategies to deal with confounding factors stated?	□	□	□	■
7. Were the outcomes measured in a valid and reliable way and expressed clearly? Has been used any reliable statistical methods to evaluate the phylogenetic results?	□	■	□	□
8. Was appropriate statistical analysis used?	■	□	□	□

Overall appraisal: Include ■ Exclude □ Seek further info □

Comments (Including reason for exclusion)

Criticality: missing data (time period), there is a partial correlation between accession numbers and subgenotypes

Ranking: good (66%)

© JBI, 2020. All rights reserved. JBI grants use of these tools for research purposes only. All other enquiries should be sent to jbisynthesis@adelaide.edu.au.

Reviewer SILVIA PAVONE/PAOLA GOBBI Date_________17/08/2023______________

Author Giammarioli et al., Year 2011 Record Number 29

	Yes	No	Unclear	Not applicable
1. Were the criteria for inclusion in the sample clearly defined?	■	□	□	□
2. Were the study subjects and the setting described in detail?	■	□	□	□
3. Was the exposure measured in a valid and reliable way?	■	□	□	□
4. Were objective, standard criteria used for measurement of the condition?	■	□	□	□
5. Were confounding factors identified?	□	□	□	■
6. Were strategies to deal with confounding factors stated?	□	□	□	■
7. Were the outcomes measured in a valid and reliable way and expressed clearly? Has been used any reliable statistical methods to evaluate the phylogenetic results?	■	□	□	□
8. Was appropriate statistical analysis used?	■	□	□	□

Overall appraisal: Include ■ Exclude □ Seek further info □

Comments (Including reason for exclusion)

Ranking: very good (100%)

© JBI, 2020. All rights reserved. JBI grants use of these tools for research purposes only. All other enquiries should be sent to jbisynthesis@adelaide.edu.au.

Reviewer SILVIA PAVONE/PAOLA GOBBI Date_________17/08/2023______________

Author Gil et al., Year 2006 Record Number 30

	Yes	No	Unclear	Not applicable
1. Were the criteria for inclusion in the sample clearly defined?	■	□	□	□
2. Were the study subjects and the setting described in detail?	□	■	□	□
3. Was the exposure measured in a valid and reliable way?	■	□	□	□
4. Were objective, standard criteria used for measurement of the condition?	■	□	□	□
5. Were confounding factors identified?	□	□	□	■
6. Were strategies to deal with confounding factors stated?	□	□	□	■
7. Were the outcomes measured in a valid and reliable way and expressed clearly? Has been used any reliable statistical methods to evaluate the phylogenetic results?	□	■	□	□
8. Was appropriate statistical analysis used?	□	■	□	□

Overall appraisal: Include ■ Exclude □ Seek further info □

Comments (Including reason for exclusion)

Criticality: missing data (time period, accession numbers, bootstrap values)

Ranking: unsatisfactory (50%)

© JBI, 2020. All rights reserved. JBI grants use of these tools for research purposes only. All other enquiries should be sent to jbisynthesis@adelaide.edu.au.

Reviewer SILVIA PAVONE/PAOLA GOBBI Date_________17/08/2023______________

Author Gjerset et al., Year 2007 Record Number 31

	Yes	No	Unclear	Not applicable
1. Were the criteria for inclusion in the sample clearly defined?	■	□	□	□
2. Were the study subjects and the setting described in detail?	■	□	□	□
3. Was the exposure measured in a valid and reliable way?	■	□	□	□
4. Were objective, standard criteria used for measurement of the condition?	■	□	□	□
5. Were confounding factors identified?	□	□	□	■
6. Were strategies to deal with confounding factors stated?	□	□	□	■
7. Were the outcomes measured in a valid and reliable way and expressed clearly? Has been used any reliable statistical methods to evaluate the phylogenetic results?	□	■	□	□
8. Was appropriate statistical analysis used?	■	□	□	□

Overall appraisal: Include ■ Exclude □ Seek further info □

Comments (Including reason for exclusion)

Criticality: missing data (sequencing technique), there is no correlation between accession numbers and subgenotypes

Ranking: very good (83%)

© JBI, 2020. All rights reserved. JBI grants use of these tools for research purposes only. All other enquiries should be sent to jbisynthesis@adelaide.edu.au.

Reviewer SILVIA PAVONE/PAOLA GOBBI Date_________17/08/2023______________

Author Gjerset et al., Year 2009 Record Number 32

	Yes	No	Unclear	Not applicable
1. Were the criteria for inclusion in the sample clearly defined?	■	□	□	□
2. Were the study subjects and the setting described in detail?	■	□	□	□
3. Was the exposure measured in a valid and reliable way?	■	□	□	□
4. Were objective, standard criteria used for measurement of the condition?	■	□	□	□
5. Were confounding factors identified?	□	□	□	■
6. Were strategies to deal with confounding factors stated?	□	□	□	■
7. Were the outcomes measured in a valid and reliable way and expressed clearly? Has been used any reliable statistical methods to evaluate the phylogenetic results?	□	■	□	□
8. Was appropriate statistical analysis used?	■	□	□	□

Overall appraisal: Include ■ Exclude □ Seek further info □

Comments (Including reason for exclusion)

Criticality: there is no correlation between accession numbers and subgenotypes

Ranking: very good (83%)

© JBI, 2020. All rights reserved. JBI grants use of these tools for research purposes only. All other enquiries should be sent to jbisynthesis@adelaide.edu.au.

Reviewer SILVIA PAVONE/PAOLA GOBBI Date__________17/08/2023_______________

Author Gjerset et al., Year 2006 Record Number 33

	Yes	No	Unclear	Not applicable
1. Were the criteria for inclusion in the sample clearly defined?	■	□	□	□
2. Were the study subjects and the setting described in detail?	□	■	□	□
3. Was the exposure measured in a valid and reliable way?	■	□	□	□
4. Were objective, standard criteria used for measurement of the condition?	■	□	□	□
5. Were confounding factors identified?	□	□	□	■
6. Were strategies to deal with confounding factors stated?	□	□	□	■
7. Were the outcomes measured in a valid and reliable way and expressed clearly? Has been used any reliable statistical methods to evaluate the phylogenetic results?	■	□	□	□
8. Was appropriate statistical analysis used?	■	□	□	□

Overall appraisal: Include ■ Exclude □ Seek further info □

Comments (Including reason for exclusion)

Criticality: missing data (time period)

Ranking: very good (83%)

© JBI, 2020. All rights reserved. JBI grants use of these tools for research purposes only. All other enquiries should be sent to jbisynthesis@adelaide.edu.au.

Reviewer SILVIA PAVONE/PAOLA GOBBI Date_________17/08/2023_____________

Author Glaria et al., Year 2009 Record Number 34

	Yes	No	Unclear	Not applicable
1. Were the criteria for inclusion in the sample clearly defined?	■	□	□	□
2. Were the study subjects and the setting described in detail?	□	■	□	□
3. Was the exposure measured in a valid and reliable way?	■	□	□	□
4. Were objective, standard criteria used for measurement of the condition?	■	□	□	□
5. Were confounding factors identified?	□	□	□	■
6. Were strategies to deal with confounding factors stated?	□	□	□	■
7. Were the outcomes measured in a valid and reliable way and expressed clearly? Has been used any reliable statistical methods to evaluate the phylogenetic results?	□	■	□	□
8. Was appropriate statistical analysis used?	■	□	□	□

Overall appraisal: Include ■ Exclude □ Seek further info □

Comments (Including reason for exclusion)

Criticality: missing data (time period, accession numbers corresponding to pol and gag-pol regions), there is no correlation between accession numbers and subgenotypes

Ranking: good (66%)

© JBI, 2020. All rights reserved. JBI grants use of these tools for research purposes only. All other enquiries should be sent to jbisynthesis@adelaide.edu.au.

Reviewer SILVIA PAVONE/PAOLA GOBBI Date_________17/08/2023______________

Author Glaria et al., Year 2012 Record Number 35

	Yes	No	Unclear	Not applicable
1. Were the criteria for inclusion in the sample clearly defined?	■	□	□	□
2. Were the study subjects and the setting described in detail?	□	■	□	□
3. Was the exposure measured in a valid and reliable way?	■	□	□	□
4. Were objective, standard criteria used for measurement of the condition?	■	□	□	□
5. Were confounding factors identified?	□	□	□	■
6. Were strategies to deal with confounding factors stated?	□	□	□	■
7. Were the outcomes measured in a valid and reliable way and expressed clearly? Has been used any reliable statistical methods to evaluate the phylogenetic results?	□	□	■	□
8. Was appropriate statistical analysis used?	■	□	□	□

Overall appraisal: Include ■ Exclude □ Seek further info □

Comments (Including reason for exclusion)

Criticality: missing data (time period), unclear correlation between accession numbers and subgenotypes, only genotype A is defined

Ranking: good (75%)

© JBI, 2020. All rights reserved. JBI grants use of these tools for research purposes only. All other enquiries should be sent to jbisynthesis@adelaide.edu.au.

Reviewer SILVIA PAVONE/PAOLA GOBBI Date_________17/08/2023______________

Author Gomez-Lucia et al., Year 2013 Record Number 36

	Yes	No	Unclear	Not applicable
1. Were the criteria for inclusion in the sample clearly defined?	■	□	□	□
2. Were the study subjects and the setting described in detail?	□	■	□	□
3. Was the exposure measured in a valid and reliable way?	■	□	□	□
4. Were objective, standard criteria used for measurement of the condition?	■	□	□	□
5. Were confounding factors identified?	□	□	□	■
6. Were strategies to deal with confounding factors stated?	□	□	□	■
7. Were the outcomes measured in a valid and reliable way and expressed clearly? Has been used any reliable statistical methods to evaluate the phylogenetic results?	□	■	□	□
8. Was appropriate statistical analysis used?	□	■	□	□

Overall appraisal: Include ■ Exclude □ Seek further info □

Comments (Including reason for exclusion)

Criticality: missing data (time period, sequencing technique), no method for phylogenetic tree has been reported, there is no correlation between accession numbers and subgenotypes

Ranking: unsatisfactory (50%)

© JBI, 2020. All rights reserved. JBI grants use of these tools for research purposes only. All other enquiries should be sent to jbisynthesis@adelaide.edu.au.

Reviewer SILVIA PAVONE/PAOLA GOBBI Date_________17/08/2023_____________

Author González Méndez et al., Year 2020 Record Number 37

	Yes	No	Unclear	Not applicable
1. Were the criteria for inclusion in the sample clearly defined?	■	□	□	□
2. Were the study subjects and the setting described in detail?	□	■	□	□
3. Was the exposure measured in a valid and reliable way?	■	□	□	□
4. Were objective, standard criteria used for measurement of the condition?	■	□	□	□
5. Were confounding factors identified?	□	□	□	■
6. Were strategies to deal with confounding factors stated?	□	□	□	■
7. Were the outcomes measured in a valid and reliable way and expressed clearly? Has been used any reliable statistical methods to evaluate the phylogenetic results?	■	□	□	□
8. Was appropriate statistical analysis used?	■	□	□	□

Overall appraisal: Include ■ Exclude □ Seek further info □

Comments (Including reason for exclusion)

Criticality: missing data (time period), there is a misspelled accession number in the phylogenetic tree (MG67515132 is probably MG675132?)

Ranking: very good (83%)

© JBI, 2020. All rights reserved. JBI grants use of these tools for research purposes only. All other enquiries should be sent to jbisynthesis@adelaide.edu.au.

Reviewer SILVIA PAVONE/PAOLA GOBBI Date_________17/08/2023______________

Author Grego et al., Year 2005 Record Number 38

	Yes	No	Unclear	Not applicable
1. Were the criteria for inclusion in the sample clearly defined?	□	■	□	□
2. Were the study subjects and the setting described in detail?	□	■	□	□
3. Was the exposure measured in a valid and reliable way?	■	□	□	□
4. Were objective, standard criteria used for measurement of the condition?	■	□	□	□
5. Were confounding factors identified?	□	□	□	■
6. Were strategies to deal with confounding factors stated?	□	□	□	■
7. Were the outcomes measured in a valid and reliable way and expressed clearly? Has been used any reliable statistical methods to evaluate the phylogenetic results?	□	■	□	□
8. Was appropriate statistical analysis used?	■	□	□	□

Overall appraisal: Include ■ Exclude □ Seek further info □

Comments (Including reason for exclusion)

Criticality: No selection criteria for cases enrolment were clearly reported, missing data (time period), no subgenotypes are expressed, only genotypes

Ranking: unsatisfactory (50%)

© JBI, 2020. All rights reserved. JBI grants use of these tools for research purposes only. All other enquiries should be sent to jbisynthesis@adelaide.edu.au.

Reviewer SILVIA PAVONE/PAOLA GOBBI Date_________17/08/2023______________

Author Grego et al., Year 2007 Record Number 39

	Yes	No	Unclear	Not applicable
1. Were the criteria for inclusion in the sample clearly defined?	■	□	□	□
2. Were the study subjects and the setting described in detail?	□	■	□	□
3. Was the exposure measured in a valid and reliable way?	■	□	□	□
4. Were objective, standard criteria used for measurement of the condition?	■	□	□	□
5. Were confounding factors identified?	□	□	□	■
6. Were strategies to deal with confounding factors stated?	□	□	□	■
7. Were the outcomes measured in a valid and reliable way and expressed clearly? Has been used any reliable statistical methods to evaluate the phylogenetic results?	□	□	■	□
8. Was appropriate statistical analysis used?	■	□	□	□

Overall appraisal: Include ■ Exclude □ Seek further info □

Comments (Including reason for exclusion)

Criticality: missing data (time period), unclear sequencing method

Ranking: good (75%)

© JBI, 2020. All rights reserved. JBI grants use of these tools for research purposes only. All other enquiries should be sent to jbisynthesis@adelaide.edu.au.

Reviewer SILVIA PAVONE/PAOLA GOBBI Date_________17/08/2023______________

Author Grego et al., Year 2002 Record Number 40

	Yes	No	Unclear	Not applicable
1. Were the criteria for inclusion in the sample clearly defined?	■	□	□	□
2. Were the study subjects and the setting described in detail?	□	■	□	□
3. Was the exposure measured in a valid and reliable way?	■	□	□	□
4. Were objective, standard criteria used for measurement of the condition?	■	□	□	□
5. Were confounding factors identified?	□	□	□	■
6. Were strategies to deal with confounding factors stated?	□	□	□	■
7. Were the outcomes measured in a valid and reliable way and expressed clearly? Has been used any reliable statistical methods to evaluate the phylogenetic results?	□	■	□	□
8. Was appropriate statistical analysis used?	■	□	□	□

Overall appraisal: Include ■ Exclude □ Seek further info □

Comments (Including reason for exclusion)

Criticality: missing data (time period), there is no correlation between accession numbers and subgenotypes

Ranking: good (66%)

© JBI, 2020. All rights reserved. JBI grants use of these tools for research purposes only. All other enquiries should be sent to jbisynthesis@adelaide.edu.au.

Reviewer SILVIA PAVONE/PAOLA GOBBI Date__________17/08/2023_______________

Author Gufler et al., Year 2008 Record Number 41

	Yes	No	Unclear	Not applicable
1. Were the criteria for inclusion in the sample clearly defined?	■	□	□	□
2. Were the study subjects and the setting described in detail?	■	□	□	□
3. Was the exposure measured in a valid and reliable way?	■	□	□	□
4. Were objective, standard criteria used for measurement of the condition?	■	□	□	□
5. Were confounding factors identified?	□	□	□	■
6. Were strategies to deal with confounding factors stated?	□	□	□	■
7. Were the outcomes measured in a valid and reliable way and expressed clearly? Has been used any reliable statistical methods to evaluate the phylogenetic results?	■	□	□	□
8. Was appropriate statistical analysis used?	■	□	□	□

Overall appraisal: Include ■ Exclude □ Seek further info □

Comments (Including reason for exclusion)

Ranking: very good (100%)

© JBI, 2020. All rights reserved. JBI grants use of these tools for research purposes only. All other enquiries should be sent to jbisynthesis@adelaide.edu.au.

Reviewer SILVIA PAVONE/PAOLA GOBBI Date_________22/08/2023______________

Author Hasegawa et al., Year 2016 Record Number 42

	Yes	No	Unclear	Not applicable
1. Were the criteria for inclusion in the sample clearly defined?	■	□	□	□
2. Were the study subjects and the setting described in detail?	□	■	□	□
3. Was the exposure measured in a valid and reliable way?	■	□	□	□
4. Were objective, standard criteria used for measurement of the condition?	■	□	□	□
5. Were confounding factors identified?	□	□	□	■
6. Were strategies to deal with confounding factors stated?	□	□	□	■
7. Were the outcomes measured in a valid and reliable way and expressed clearly? Has been used any reliable statistical methods to evaluate the phylogenetic results?	□	■	□	□
8. Was appropriate statistical analysis used?	■	□	□	□

Overall appraisal: Include ■ Exclude □ Seek further info □

Comments (Including reason for exclusion)

Criticality: missing data (time period), there is a partial correlation between accession numbers and subgenotypes

Ranking: good (66%)

© JBI, 2020. All rights reserved. JBI grants use of these tools for research purposes only. All other enquiries should be sent to jbisynthesis@adelaide.edu.au.

Reviewer SILVIA PAVONE/PAOLA GOBBI Date_________22/08/2023______________

Author Herrmann et al., Year 2004 Record Number 43

	Yes	No	Unclear	Not applicable
1. Were the criteria for inclusion in the sample clearly defined?	■	□	□	□
2. Were the study subjects and the setting described in detail?	□	■	□	□
3. Was the exposure measured in a valid and reliable way?	■	□	□	□
4. Were objective, standard criteria used for measurement of the condition?	■	□	□	□
5. Were confounding factors identified?	□	□	□	■
6. Were strategies to deal with confounding factors stated?	□	□	□	■
7. Were the outcomes measured in a valid and reliable way and expressed clearly? Has been used any reliable statistical methods to evaluate the phylogenetic results?	□	■	□	□
8. Was appropriate statistical analysis used?	■	□	□	□

Overall appraisal: Include ■ Exclude □ Seek further info □

Comments (Including reason for exclusion)

Criticality: missing data (time period), there is no correlation between accession numbers and subgenotypes

Ranking: good (66%)

© JBI, 2020. All rights reserved. JBI grants use of these tools for research purposes only. All other enquiries should be sent to jbisynthesis@adelaide.edu.au.

Reviewer SILVIA PAVONE/PAOLA GOBBI Date_________22/08/2023______________

Author Herrmann-Hoesing et al., Year 2010 Record Number 44

	Yes	No	Unclear	Not applicable
1. Were the criteria for inclusion in the sample clearly defined?	■	□	□	□
2. Were the study subjects and the setting described in detail?	□	■	□	□
3. Was the exposure measured in a valid and reliable way?	■	□	□	□
4. Were objective, standard criteria used for measurement of the condition?	■	□	□	□
5. Were confounding factors identified?	□	□	□	■
6. Were strategies to deal with confounding factors stated?	□	□	□	■
7. Were the outcomes measured in a valid and reliable way and expressed clearly? Has been used any reliable statistical methods to evaluate the phylogenetic results?	□	■	□	□
8. Was appropriate statistical analysis used?	■	□	□	□

Overall appraisal: Include ■ Exclude □ Seek further info □

Comments (Including reason for exclusion)

Criticality: missing data (time period), there is no correlation between accession numbers and subgenotypes

Ranking: good (66%)

© JBI, 2020. All rights reserved. JBI grants use of these tools for research purposes only. All other enquiries should be sent to jbisynthesis@adelaide.edu.au.

Reviewer SILVIA PAVONE/PAOLA GOBBI Date_________22/08/2023_____________

Author Karr et al., Year 1996 Record Number 45

	Yes	No	Unclear	Not applicable
1. Were the criteria for inclusion in the sample clearly defined?	■	□	□	□
2. Were the study subjects and the setting described in detail?	□	■	□	□
3. Was the exposure measured in a valid and reliable way?	■	□	□	□
4. Were objective, standard criteria used for measurement of the condition?	■	□	□	□
5. Were confounding factors identified?	□	□	□	■
6. Were strategies to deal with confounding factors stated?	□	□	□	■
7. Were the outcomes measured in a valid and reliable way and expressed clearly? Has been used any reliable statistical methods to evaluate the phylogenetic results?	■	□	□	□
8. Was appropriate statistical analysis used?	■	□	□	□

Overall appraisal: Include ■ Exclude □ Seek further info □

Comments (Including reason for exclusion)

Criticality: missing data (time period)

Ranking: very good (83%)

© JBI, 2020. All rights reserved. JBI grants use of these tools for research purposes only. All other enquiries should be sent to jbisynthesis@adelaide.edu.au.

Reviewer SILVIA PAVONE/PAOLA GOBBI Date_________22/08/2023______________

Author Kokawa et al., Year 2017 Record Number 46

	Yes	No	Unclear	Not applicable
1. Were the criteria for inclusion in the sample clearly defined?	■	□	□	□
2. Were the study subjects and the setting described in detail?	■	□	□	□
3. Was the exposure measured in a valid and reliable way?	■	□	□	□
4. Were objective, standard criteria used for measurement of the condition?	■	□	□	□
5. Were confounding factors identified?	□	□	□	■
6. Were strategies to deal with confounding factors stated?	□	□	□	■
7. Were the outcomes measured in a valid and reliable way and expressed clearly? Has been used any reliable statistical methods to evaluate the phylogenetic results?	■	□	□	□
8. Was appropriate statistical analysis used?	■	□	□	□

Overall appraisal: Include ■ Exclude □ Seek further info □

Comments (Including reason for exclusion)

Ranking: very good (100%)

© JBI, 2020. All rights reserved. JBI grants use of these tools for research purposes only. All other enquiries should be sent to jbisynthesis@adelaide.edu.au.

Reviewer SILVIA PAVONE/PAOLA GOBBI Date_________22/08/2023______________

Author Kuhar et al., Year 2013 Record Number 47

	Yes	No	Unclear	Not applicable
1. Were the criteria for inclusion in the sample clearly defined?	■	□	□	□
2. Were the study subjects and the setting described in detail?	□	■	□	□
3. Was the exposure measured in a valid and reliable way?	■	□	□	□
4. Were objective, standard criteria used for measurement of the condition?	■	□	□	□
5. Were confounding factors identified?	□	□	□	■
6. Were strategies to deal with confounding factors stated?	□	□	□	■
7. Were the outcomes measured in a valid and reliable way and expressed clearly? Has been used any reliable statistical methods to evaluate the phylogenetic results?	□	■	□	□
8. Was appropriate statistical analysis used?	■	□	□	□

Overall appraisal: Include ■ Exclude □ Seek further info □

Comments (Including reason for exclusion)

Criticality: missing data (time period), there is no correlation between accession numbers and subgenotypes

Ranking: good (66%)

© JBI, 2020. All rights reserved. JBI grants use of these tools for research purposes only. All other enquiries should be sent to jbisynthesis@adelaide.edu.au.

Reviewer SILVIA PAVONE/PAOLA GOBBI Date_________22/08/2023______________

Author Kuhar et al., Year 2013 Record Number 48

	Yes	No	Unclear	Not applicable
1. Were the criteria for inclusion in the sample clearly defined?	■	□	□	□
2. Were the study subjects and the setting described in detail?	□	■	□	□
3. Was the exposure measured in a valid and reliable way?	■	□	□	□
4. Were objective, standard criteria used for measurement of the condition?	■	□	□	□
5. Were confounding factors identified?	□	□	□	■
6. Were strategies to deal with confounding factors stated?	□	□	□	■
7. Were the outcomes measured in a valid and reliable way and expressed clearly? Has been used any reliable statistical methods to evaluate the phylogenetic results?	□	■	□	□
8. Was appropriate statistical analysis used?	■	□	□	□

Overall appraisal: Include ■ Exclude □ Seek further info □

Comments (Including reason for exclusion)

Criticality: missing data (time period), there is no correlation between accession numbers and subgenotypes

Ranking: good (66%)

© JBI, 2020. All rights reserved. JBI grants use of these tools for research purposes only. All other enquiries should be sent to jbisynthesis@adelaide.edu.au.

Reviewer SILVIA PAVONE/PAOLA GOBBI Date_________22/08/2023______________

Author Kuzmak et al., Year 2007 Record Number 49

	Yes	No	Unclear	Not applicable
1. Were the criteria for inclusion in the sample clearly defined?	■	□	□	□
2. Were the study subjects and the setting described in detail?	□	■	□	□
3. Was the exposure measured in a valid and reliable way?	■	□	□	□
4. Were objective, standard criteria used for measurement of the condition?	■	□	□	□
5. Were confounding factors identified?	□	□	□	■
6. Were strategies to deal with confounding factors stated?	□	□	□	■
7. Were the outcomes measured in a valid and reliable way and expressed clearly? Has been used any reliable statistical methods to evaluate the phylogenetic results?	□	□	■	□
8. Was appropriate statistical analysis used?	■	□	□	□

Overall appraisal: Include ■ Exclude □ Seek further info □

Comments (Including reason for exclusion)

Criticality: missing data (time period), unclear bootstrap values

Ranking: good (75%)

© JBI, 2020. All rights reserved. JBI grants use of these tools for research purposes only. All other enquiries should be sent to jbisynthesis@adelaide.edu.au.

Reviewer SILVIA PAVONE/PAOLA GOBBI Date_________22/08/2023______________

Author Laamanen et al., Year 2007 Record Number 50

	Yes	No	Unclear	Not applicable
1. Were the criteria for inclusion in the sample clearly defined?	■	□	□	□
2. Were the study subjects and the setting described in detail?	□	■	□	□
3. Was the exposure measured in a valid and reliable way?	■	□	□	□
4. Were objective, standard criteria used for measurement of the condition?	■	□	□	□
5. Were confounding factors identified?	□	□	□	■
6. Were strategies to deal with confounding factors stated?	□	□	□	■
7. Were the outcomes measured in a valid and reliable way and expressed clearly? Has been used any reliable statistical methods to evaluate the phylogenetic results?	□	■	□	□
8. Was appropriate statistical analysis used?	■	□	□	□

Overall appraisal: Include ■ Exclude □ Seek further info □

Comments (Including reason for exclusion)

Criticality: missing data (time period), there is no correlation between accession numbers and subgenotypes

Ranking: good (66%)

© JBI, 2020. All rights reserved. JBI grants use of these tools for research purposes only. All other enquiries should be sent to jbisynthesis@adelaide.edu.au.

Reviewer SILVIA PAVONE/PAOLA GOBBI Date__________22/08/2023________________

Author Leroux et al., Year 1995 Record Number 51

	Yes	No	Unclear	Not applicable
1. Were the criteria for inclusion in the sample clearly defined?	■	□	□	□
2. Were the study subjects and the setting described in detail?	□	■	□	□
3. Was the exposure measured in a valid and reliable way?	■	□	□	□
4. Were objective, standard criteria used for measurement of the condition?	■	□	□	□
5. Were confounding factors identified?	□	□	□	■
6. Were strategies to deal with confounding factors stated?	□	□	□	■
7. Were the outcomes measured in a valid and reliable way and expressed clearly? Has been used any reliable statistical methods to evaluate the phylogenetic results?	□	■	□	□
8. Was appropriate statistical analysis used?	■	□	□	□

Overall appraisal: Include ■ Exclude □ Seek further info □

Comments (Including reason for exclusion)

Criticality: missing data (time period), there is no correlation between accession numbers and subgenotypes or accession numbers and genomic regions

Ranking: good (66%)

© JBI, 2020. All rights reserved. JBI grants use of these tools for research purposes only. All other enquiries should be sent to jbisynthesis@adelaide.edu.au.

Reviewer SILVIA PAVONE/PAOLA GOBBI Date_________19/09/2023______________

Author L'Homme et al., Year 2015 Record Number 52

	Yes	No	Unclear	Not applicable
1. Were the criteria for inclusion in the sample clearly defined?	■	□	□	□
2. Were the study subjects and the setting described in detail?	■	□	□	□
3. Was the exposure measured in a valid and reliable way?	■	□	□	□
4. Were objective, standard criteria used for measurement of the condition?	■	□	□	□
5. Were confounding factors identified?	□	□	□	■
6. Were strategies to deal with confounding factors stated?	□	□	□	■
7. Were the outcomes measured in a valid and reliable way and expressed clearly? Has been used any reliable statistical methods to evaluate the phylogenetic results?	□	■	□	□
8. Was appropriate statistical analysis used?	■	□	□	□

Overall appraisal: Include ■ Exclude □ Seek further info □

Comments (Including reason for exclusion)

Criticality: there is no correlation between accession numbers and subgenotypes

Ranking: very good (83%)

© JBI, 2020. All rights reserved. JBI grants use of these tools for research purposes only. All other enquiries should be sent to jbisynthesis@adelaide.edu.au.

Reviewer SILVIA PAVONE/PAOLA GOBBI Date_________19/09/2023_____________

Author L'Homme et al., Year 2011 Record Number 53

	Yes	No	Unclear	Not applicable
1. Were the criteria for inclusion in the sample clearly defined?	■	□	□	□
2. Were the study subjects and the setting described in detail?	■	□	□	□
3. Was the exposure measured in a valid and reliable way?	■	□	□	□
4. Were objective, standard criteria used for measurement of the condition?	■	□	□	□
5. Were confounding factors identified?	□	□	□	■
6. Were strategies to deal with confounding factors stated?	□	□	□	■
7. Were the outcomes measured in a valid and reliable way and expressed clearly? Has been used any reliable statistical methods to evaluate the phylogenetic results?	□	■	□	□
8. Was appropriate statistical analysis used?	■	□	□	□

Overall appraisal: Include ■ Exclude □ Seek further info □

Comments (Including reason for exclusion)

Criticality: there is no correlation between accession numbers and subgenotypes

Ranking: very good (83%)

© JBI, 2020. All rights reserved. JBI grants use of these tools for research purposes only. All other enquiries should be sent to jbisynthesis@adelaide.edu.au.

Reviewer SILVIA PAVONE/PAOLA GOBBI Date__________19/09/2023_______________

Author Marinho et al., Year 2018 Record Number 54

	Yes	No	Unclear	Not applicable
1. Were the criteria for inclusion in the sample clearly defined?	■	□	□	□
2. Were the study subjects and the setting described in detail?	□	■	□	□
3. Was the exposure measured in a valid and reliable way?	■	□	□	□
4. Were objective, standard criteria used for measurement of the condition?	■	□	□	□
5. Were confounding factors identified?	□	□	□	■
6. Were strategies to deal with confounding factors stated?	□	□	□	■
7. Were the outcomes measured in a valid and reliable way and expressed clearly? Has been used any reliable statistical methods to evaluate the phylogenetic results?	■	□	□	□
8. Was appropriate statistical analysis used?	■	□	□	□

Overall appraisal: Include ■ Exclude □ Seek further info □

Comments (Including reason for exclusion)

Criticality: missing time period

Ranking: very good (83%)

© JBI, 2020. All rights reserved. JBI grants use of these tools for research purposes only. All other enquiries should be sent to jbisynthesis@adelaide.edu.au.

Reviewer SILVIA PAVONE/PAOLA GOBBI Date_________19/09/2023______________

Author Mendiola et al., Year 2019 Record Number 55

	Yes	No	Unclear	Not applicable
1. Were the criteria for inclusion in the sample clearly defined?	■	□	□	□
2. Were the study subjects and the setting described in detail?	□	■	□	□
3. Was the exposure measured in a valid and reliable way?	■	□	□	□
4. Were objective, standard criteria used for measurement of the condition?	■	□	□	□
5. Were confounding factors identified?	□	□	□	■
6. Were strategies to deal with confounding factors stated?	□	□	□	■
7. Were the outcomes measured in a valid and reliable way and expressed clearly? Has been used any reliable statistical methods to evaluate the phylogenetic results?	■	□	□	□
8. Was appropriate statistical analysis used?	■	□	□	□

Overall appraisal: Include ■ Exclude □ Seek further info □

Comments (Including reason for exclusion)

Criticality: missing time period

Ranking: very good (83%)

© JBI, 2020. All rights reserved. JBI grants use of these tools for research purposes only. All other enquiries should be sent to jbisynthesis@adelaide.edu.au.

Reviewer SILVIA PAVONE/PAOLA GOBBI Date__________19/09/2023_______________

Author Michiels et al., Year 2020 Record Number 56

	Yes	No	Unclear	Not applicable
1. Were the criteria for inclusion in the sample clearly defined?	■	□	□	□
2. Were the study subjects and the setting described in detail?	□	■	□	□
3. Was the exposure measured in a valid and reliable way?	■	□	□	□
4. Were objective, standard criteria used for measurement of the condition?	■	□	□	□
5. Were confounding factors identified?	□	□	□	■
6. Were strategies to deal with confounding factors stated?	□	□	□	■
7. Were the outcomes measured in a valid and reliable way and expressed clearly? Has been used any reliable statistical methods to evaluate the phylogenetic results?	■	□	□	□
8. Was appropriate statistical analysis used?	■	□	□	□

Overall appraisal: Include ■ Exclude □ Seek further info □

Comments (Including reason for exclusion)

Criticality: missing time period

Ranking: very good (83%)

© JBI, 2020. All rights reserved. JBI grants use of these tools for research purposes only. All other enquiries should be sent to jbisynthesis@adelaide.edu.au.

Reviewer SILVIA PAVONE/PAOLA GOBBI Date_________19/09/2023______________

Author Molaee et al., Year 2020 Record Number 57

	Yes	No	Unclear	Not applicable
1. Were the criteria for inclusion in the sample clearly defined?	■	□	□	□
2. Were the study subjects and the setting described in detail?	□	■	□	□
3. Was the exposure measured in a valid and reliable way?	■	□	□	□
4. Were objective, standard criteria used for measurement of the condition?	■	□	□	□
5. Were confounding factors identified?	□	□	□	■
6. Were strategies to deal with confounding factors stated?	□	□	□	■
7. Were the outcomes measured in a valid and reliable way and expressed clearly? Has been used any reliable statistical methods to evaluate the phylogenetic results?	■	□	□	□
8. Was appropriate statistical analysis used?	■	□	□	□

Overall appraisal: Include ■ Exclude □ Seek further info □

Comments (Including reason for exclusion)

Criticality: missing time period

Ranking: very good (83%)

© JBI, 2020. All rights reserved. JBI grants use of these tools for research purposes only. All other enquiries should be sent to jbisynthesis@adelaide.edu.au.

Reviewer SILVIA PAVONE/PAOLA GOBBI Date__________19/09/2023_______________

Author Mosa et al., Year 2022 Record Number 58

	Yes	No	Unclear	Not applicable
1. Were the criteria for inclusion in the sample clearly defined?	■	□	□	□
2. Were the study subjects and the setting described in detail?	■	□	□	□
3. Was the exposure measured in a valid and reliable way?	■	□	□	□
4. Were objective, standard criteria used for measurement of the condition?	■	□	□	□
5. Were confounding factors identified?	□	□	□	■
6. Were strategies to deal with confounding factors stated?	□	□	□	■
7. Were the outcomes measured in a valid and reliable way and expressed clearly? Has been used any reliable statistical methods to evaluate the phylogenetic results?	□	■	□	□
8. Was appropriate statistical analysis used?	■	□	□	□

Overall appraisal: Include ■ Exclude □ Seek further info □

Comments (Including reason for exclusion)

Criticality: missing data (sequencing method), there is no correlation between accession numbers and genotypes

Ranking: very good (83%)

© JBI, 2020. All rights reserved. JBI grants use of these tools for research purposes only. All other enquiries should be sent to jbisynthesis@adelaide.edu.au.

Reviewer SILVIA PAVONE/PAOLA GOBBI Date_________19/09/2023______________

Author Muz et al., Year 2013 Record Number 59

	Yes	No	Unclear	Not applicable
1. Were the criteria for inclusion in the sample clearly defined?	■	□	□	□
2. Were the study subjects and the setting described in detail?	■	□	□	□
3. Was the exposure measured in a valid and reliable way?	■	□	□	□
4. Were objective, standard criteria used for measurement of the condition?	■	□	□	□
5. Were confounding factors identified?	□	□	□	■
6. Were strategies to deal with confounding factors stated?	□	□	□	■
7. Were the outcomes measured in a valid and reliable way and expressed clearly? Has been used any reliable statistical methods to evaluate the phylogenetic results?	□	■	□	□
8. Was appropriate statistical analysis used?	■	□	□	□

Overall appraisal: Include ■ Exclude □ Seek further info □

Comments (Including reason for exclusion)

Criticality: there is no correlation between accession numbers and genotypes, low bootstrap values

Ranking: very good (83%)

© JBI, 2020. All rights reserved. JBI grants use of these tools for research purposes only. All other enquiries should be sent to jbisynthesis@adelaide.edu.au.

Reviewer SILVIA PAVONE/PAOLA GOBBI Date_________19/09/2023______________

Author Mwaengo et al., Year 1997 Record Number 60

	Yes	No	Unclear	Not applicable
1. Were the criteria for inclusion in the sample clearly defined?	■	□	□	□
2. Were the study subjects and the setting described in detail?	□	■	□	□
3. Was the exposure measured in a valid and reliable way?	■	□	□	□
4. Were objective, standard criteria used for measurement of the condition?	□	■	□	□
5. Were confounding factors identified?	□	□	□	■
6. Were strategies to deal with confounding factors stated?	□	□	□	■
7. Were the outcomes measured in a valid and reliable way and expressed clearly? Has been used any reliable statistical methods to evaluate the phylogenetic results?	□	■	□	□
8. Was appropriate statistical analysis used?	■	□	□	□

Overall appraisal: Include ■ Exclude □ Seek further info □

Comments (Including reason for exclusion)

Criticality: missing data (time period), there is no correlation between accession numbers and genotypes, pol genomic length not present

Ranking: unsatisfactory (50%)

© JBI, 2020. All rights reserved. JBI grants use of these tools for research purposes only. All other enquiries should be sent to jbisynthesis@adelaide.edu.au.

Reviewer SILVIA PAVONE/PAOLA GOBBI Date_________19/09/2023______________

Author Oguma et al., Year 2013 Record Number 61

	Yes	No	Unclear	Not applicable
1. Were the criteria for inclusion in the sample clearly defined?	■	□	□	□
2. Were the study subjects and the setting described in detail?	□	■	□	□
3. Was the exposure measured in a valid and reliable way?	■	□	□	□
4. Were objective, standard criteria used for measurement of the condition?	■	□	□	□
5. Were confounding factors identified?	□	□	□	■
6. Were strategies to deal with confounding factors stated?	□	□	□	■
7. Were the outcomes measured in a valid and reliable way and expressed clearly? Has been used any reliable statistical methods to evaluate the phylogenetic results?	■	□	□	□
8. Was appropriate statistical analysis used?	■	□	□	□

Overall appraisal: Include ■ Exclude □ Seek further info □

Comments (Including reason for exclusion)

Criticality: missing time period

Ranking: very good (83%)

© JBI, 2020. All rights reserved. JBI grants use of these tools for research purposes only. All other enquiries should be sent to jbisynthesis@adelaide.edu.au.

Reviewer SILVIA PAVONE/PAOLA GOBBI Date_________19/09/2023______________

Author Olech et al., Year 2021 Record Number 62

	Yes	No	Unclear	Not applicable
1. Were the criteria for inclusion in the sample clearly defined?	■	□	□	□
2. Were the study subjects and the setting described in detail?	□	■	□	□
3. Was the exposure measured in a valid and reliable way?	■	□	□	□
4. Were objective, standard criteria used for measurement of the condition?	■	□	□	□
5. Were confounding factors identified?	□	□	□	■
6. Were strategies to deal with confounding factors stated?	□	□	□	■
7. Were the outcomes measured in a valid and reliable way and expressed clearly? Has been used any reliable statistical methods to evaluate the phylogenetic results?	□	■	□	□
8. Was appropriate statistical analysis used?	■	□	□	□

Overall appraisal: Include ■ Exclude □ Seek further info □

Comments (Including reason for exclusion)

Criticality: missing data (time period), there is no correlation between accession numbers and genotypes, it is not specified which samples have been sequenced by NG techniques

Ranking: good (66%)

© JBI, 2020. All rights reserved. JBI grants use of these tools for research purposes only. All other enquiries should be sent to jbisynthesis@adelaide.edu.au.

Reviewer SILVIA PAVONE/PAOLA GOBBI Date_________19/09/2023______________

Author Olech et al., Year 2019 Record Number 63

	Yes	No	Unclear	Not applicable
1. Were the criteria for inclusion in the sample clearly defined?	■	□	□	□
2. Were the study subjects and the setting described in detail?	□	■	□	□
3. Was the exposure measured in a valid and reliable way?	■	□	□	□
4. Were objective, standard criteria used for measurement of the condition?	■	□	□	□
5. Were confounding factors identified?	□	□	□	■
6. Were strategies to deal with confounding factors stated?	□	□	□	■
7. Were the outcomes measured in a valid and reliable way and expressed clearly? Has been used any reliable statistical methods to evaluate the phylogenetic results?	□	■	□	□
8. Was appropriate statistical analysis used?	■	□	□	□

Overall appraisal: Include ■ Exclude □ Seek further info □

Comments (Including reason for exclusion)

Criticality: missing data (time period), there is no correlation between accession numbers and genotypes

Ranking: good (66%)

© JBI, 2020. All rights reserved. JBI grants use of these tools for research purposes only. All other enquiries should be sent to jbisynthesis@adelaide.edu.au.

Reviewer SILVIA PAVONE/PAOLA GOBBI Date_________19/09/2023______________

Author Olech et al., Year 2020 Record Number 64

	Yes	No	Unclear	Not applicable
1. Were the criteria for inclusion in the sample clearly defined?	■	□	□	□
2. Were the study subjects and the setting described in detail?	■	□	□	□
3. Was the exposure measured in a valid and reliable way?	■	□	□	□
4. Were objective, standard criteria used for measurement of the condition?	■	□	□	□
5. Were confounding factors identified?	□	□	□	■
6. Were strategies to deal with confounding factors stated?	□	□	□	■
7. Were the outcomes measured in a valid and reliable way and expressed clearly? Has been used any reliable statistical methods to evaluate the phylogenetic results?	■	□	□	□
8. Was appropriate statistical analysis used?	■	□	□	□

Overall appraisal: Include ■ Exclude □ Seek further info □

Comments (Including reason for exclusion)

Ranking: very good (100%)

© JBI, 2020. All rights reserved. JBI grants use of these tools for research purposes only. All other enquiries should be sent to jbisynthesis@adelaide.edu.au.

Reviewer SILVIA PAVONE/PAOLA GOBBI Date_________19/09/2023______________

Author Olech et al., Year 2021 Record Number 65

	Yes	No	Unclear	Not applicable
1. Were the criteria for inclusion in the sample clearly defined?	■	□	□	□
2. Were the study subjects and the setting described in detail?	□	■	□	□
3. Was the exposure measured in a valid and reliable way?	■	□	□	□
4. Were objective, standard criteria used for measurement of the condition?	■	□	□	□
5. Were confounding factors identified?	□	□	□	■
6. Were strategies to deal with confounding factors stated?	□	□	□	■
7. Were the outcomes measured in a valid and reliable way and expressed clearly? Has been used any reliable statistical methods to evaluate the phylogenetic results?	■	□	□	□
8. Was appropriate statistical analysis used?	■	□	□	□

Overall appraisal: Include ■ Exclude □ Seek further info □

Comments (Including reason for exclusion)

Criticality: missing data (time period)

Ranking: very good (83%)

© JBI, 2020. All rights reserved. JBI grants use of these tools for research purposes only. All other enquiries should be sent to jbisynthesis@adelaide.edu.au.

Reviewer SILVIA PAVONE/PAOLA GOBBI Date_________19/09/2023______________

Author Olech et al., Year 2022 Record Number 66

	Yes	No	Unclear	Not applicable
1. Were the criteria for inclusion in the sample clearly defined?	■	□	□	□
2. Were the study subjects and the setting described in detail?	■	□	□	□
3. Was the exposure measured in a valid and reliable way?	■	□	□	□
4. Were objective, standard criteria used for measurement of the condition?	■	□	□	□
5. Were confounding factors identified?	□	□	□	■
6. Were strategies to deal with confounding factors stated?	□	□	□	■
7. Were the outcomes measured in a valid and reliable way and expressed clearly? Has been used any reliable statistical methods to evaluate the phylogenetic results?	□	■	□	□
8. Was appropriate statistical analysis used?	■	□	□	□

Overall appraisal: Include ■ Exclude □ Seek further info □

Comments (Including reason for exclusion)

Criticality: low bootstrap values

Ranking: very good (83%)

© JBI, 2020. All rights reserved. JBI grants use of these tools for research purposes only. All other enquiries should be sent to jbisynthesis@adelaide.edu.au.

Reviewer SILVIA PAVONE/PAOLA GOBBI Date_________20/09/2023______________

Author Olech et al., Year 2022 Record Number 67

	Yes	No	Unclear	Not applicable
1. Were the criteria for inclusion in the sample clearly defined?	■	☐	☐	☐
2. Were the study subjects and the setting described in detail?	☐	■	☐	☐
3. Was the exposure measured in a valid and reliable way?	■	☐	☐	☐
4. Were objective, standard criteria used for measurement of the condition?	■	☐	☐	☐
5. Were confounding factors identified?	☐	☐	☐	■
6. Were strategies to deal with confounding factors stated?	☐	☐	☐	■
7. Were the outcomes measured in a valid and reliable way and expressed clearly? Has been used any reliable statistical methods to evaluate the phylogenetic results?	■	☐	☐	☐
8. Was appropriate statistical analysis used?	■	☐	☐	☐

Overall appraisal: Include ■ Exclude ☐ Seek further info ☐

Comments (Including reason for exclusion)

Criticality: missing data (time period)

Ranking: very good (83%)

© JBI, 2020. All rights reserved. JBI grants use of these tools for research purposes only. All other enquiries should be sent to jbisynthesis@adelaide.edu.au.

Reviewer SILVIA PAVONE/PAOLA GOBBI Date_________20/09/2023______________

Author Olech et al., Year 2019 Record Number 68

	Yes	No	Unclear	Not applicable
1. Were the criteria for inclusion in the sample clearly defined?	■	□	□	□
2. Were the study subjects and the setting described in detail?	□	■	□	□
3. Was the exposure measured in a valid and reliable way?	■	□	□	□
4. Were objective, standard criteria used for measurement of the condition?	■	□	□	□
5. Were confounding factors identified?	□	□	□	■
6. Were strategies to deal with confounding factors stated?	□	□	□	■
7. Were the outcomes measured in a valid and reliable way and expressed clearly? Has been used any reliable statistical methods to evaluate the phylogenetic results?	□	■	□	□
8. Was appropriate statistical analysis used?	■	□	□	□

Overall appraisal: Include ■ Exclude □ Seek further info □

Comments (Including reason for exclusion)

Criticality: missing data (time period), there is no correlation between accession numbers and genotypes

Ranking: good (66%)

© JBI, 2020. All rights reserved. JBI grants use of these tools for research purposes only. All other enquiries should be sent to jbisynthesis@adelaide.edu.au.

Reviewer SILVIA PAVONE/PAOLA GOBBI Date_________20/09/2023______________

Author Olech et al., Year 2012 Record Number 69

	Yes	No	Unclear	Not applicable
1. Were the criteria for inclusion in the sample clearly defined?	■	□	□	□
2. Were the study subjects and the setting described in detail?	□	■	□	□
3. Was the exposure measured in a valid and reliable way?	■	□	□	□
4. Were objective, standard criteria used for measurement of the condition?	■	□	□	□
5. Were confounding factors identified?	□	□	□	■
6. Were strategies to deal with confounding factors stated?	□	□	□	■
7. Were the outcomes measured in a valid and reliable way and expressed clearly? Has been used any reliable statistical methods to evaluate the phylogenetic results?	□	■	□	□
8. Was appropriate statistical analysis used?	■	□	□	□

Overall appraisal: Include ■ Exclude □ Seek further info □

Comments (Including reason for exclusion)

Criticality: missing data (time period), there is no correlation between accession numbers and genotypes

Ranking: good (66%)

© JBI, 2020. All rights reserved. JBI grants use of these tools for research purposes only. All other enquiries should be sent to jbisynthesis@adelaide.edu.au.

Reviewer SILVIA PAVONE/PAOLA GOBBI Date_________20/09/2023______________

Author Olech et al., Year 2018 Record Number 70

	Yes	No	Unclear	Not applicable
1. Were the criteria for inclusion in the sample clearly defined?	■	□	□	□
2. Were the study subjects and the setting described in detail?	□	■	□	□
3. Was the exposure measured in a valid and reliable way?	■	□	□	□
4. Were objective, standard criteria used for measurement of the condition?	■	□	□	□
5. Were confounding factors identified?	□	□	□	■
6. Were strategies to deal with confounding factors stated?	□	□	□	■
7. Were the outcomes measured in a valid and reliable way and expressed clearly? Has been used any reliable statistical methods to evaluate the phylogenetic results?	□	■	□	□
8. Was appropriate statistical analysis used?	■	□	□	□

Overall appraisal: Include ■ Exclude □ Seek further info □

Comments (Including reason for exclusion)

Criticality: missing data (time period), there is no correlation between accession numbers and genotypes

Ranking: good (66%)

© JBI, 2020. All rights reserved. JBI grants use of these tools for research purposes only. All other enquiries should be sent to jbisynthesis@adelaide.edu.au.

Reviewer SILVIA PAVONE/PAOLA GOBBI Date_________20/09/2023______________

Author Padiernos et al., Year 2015 Record Number 71

	Yes	No	Unclear	Not applicable
1. Were the criteria for inclusion in the sample clearly defined?	■	□	□	□
2. Were the study subjects and the setting described in detail?	□	■	□	□
3. Was the exposure measured in a valid and reliable way?	■	□	□	□
4. Were objective, standard criteria used for measurement of the condition?	□	□	■	□
5. Were confounding factors identified?	□	□	□	■
6. Were strategies to deal with confounding factors stated?	□	□	□	■
7. Were the outcomes measured in a valid and reliable way and expressed clearly? Has been used any reliable statistical methods to evaluate the phylogenetic results?	□	□	■	□
8. Was appropriate statistical analysis used?	■	□	□	□

Overall appraisal: Include ■ Exclude □ Seek further info □

Comments (Including reason for exclusion)

Criticality: missing data (time period, accession numbers), the paper is unclear about the bp-length of sequenced fragments (there are differences between length reported in M&M and length reported in phylogenetic tree)

Ranking: good (66%)

© JBI, 2020. All rights reserved. JBI grants use of these tools for research purposes only. All other enquiries should be sent to jbisynthesis@adelaide.edu.au.

Reviewer SILVIA PAVONE/PAOLA GOBBI Date_________20/09/2023______________

Author Panei et al., Year 2017 Record Number 72

	Yes	No	Unclear	Not applicable
1. Were the criteria for inclusion in the sample clearly defined?	■	□	□	□
2. Were the study subjects and the setting described in detail?	□	■	□	□
3. Was the exposure measured in a valid and reliable way?	■	□	□	□
4. Were objective, standard criteria used for measurement of the condition?	□	□	■	□
5. Were confounding factors identified?	□	□	□	■
6. Were strategies to deal with confounding factors stated?	□	□	□	■
7. Were the outcomes measured in a valid and reliable way and expressed clearly? Has been used any reliable statistical methods to evaluate the phylogenetic results?	□	□	■	□
8. Was appropriate statistical analysis used?	■	□	□	□

Overall appraisal: Include ■ Exclude □ Seek further info □

Comments (Including reason for exclusion)

Criticality: missing data (time period), it is unclear whether they have sequenced the complete gag gene or not, it is odd that they compare in the same phylogenetic tree a partial sequence (645 bp) with complete genomes (ca 9000 bp) and complete gag (1300 bp)

Ranking: good (66%)

© JBI, 2020. All rights reserved. JBI grants use of these tools for research purposes only. All other enquiries should be sent to jbisynthesis@adelaide.edu.au.

Reviewer SILVIA PAVONE/PAOLA GOBBI Date_________20/09/2023______________

Author Park et al., Year 2010 Record Number 73

	Yes	No	Unclear	Not applicable
1. Were the criteria for inclusion in the sample clearly defined?	■	□	□	□
2. Were the study subjects and the setting described in detail?	□	■	□	□
3. Was the exposure measured in a valid and reliable way?	■	□	□	□
4. Were objective, standard criteria used for measurement of the condition?	■	□	□	□
5. Were confounding factors identified?	□	□	□	■
6. Were strategies to deal with confounding factors stated?	□	□	□	■
7. Were the outcomes measured in a valid and reliable way and expressed clearly? Has been used any reliable statistical methods to evaluate the phylogenetic results?	□	■	□	□
8. Was appropriate statistical analysis used?	■	□	□	□

Overall appraisal: Include ■ Exclude □ Seek further info □

Comments (Including reason for exclusion)

Criticality: missing data (time period, accession numbers), short genomic region used, low bootstrap values in tree

Ranking: good (66%)

© JBI, 2020. All rights reserved. JBI grants use of these tools for research purposes only. All other enquiries should be sent to jbisynthesis@adelaide.edu.au.

Reviewer SILVIA PAVONE/PAOLA GOBBI Date_________25/09/2023______________

Author Pisoni et al., Year 2006 Record Number 74

	Yes	No	Unclear	Not applicable
1. Were the criteria for inclusion in the sample clearly defined?	■	□	□	□
2. Were the study subjects and the setting described in detail?	□	■	□	□
3. Was the exposure measured in a valid and reliable way?	■	□	□	□
4. Were objective, standard criteria used for measurement of the condition?	■	□	□	□
5. Were confounding factors identified?	□	□	□	■
6. Were strategies to deal with confounding factors stated?	□	□	□	■
7. Were the outcomes measured in a valid and reliable way and expressed clearly? Has been used any reliable statistical methods to evaluate the phylogenetic results?	■	□	□	□
8. Was appropriate statistical analysis used?	■	□	□	□

Overall appraisal: Include ■ Exclude □ Seek further info □

Comments (Including reason for exclusion)

Criticality: missing data (time period)

Ranking: very good (83%)

© JBI, 2020. All rights reserved. JBI grants use of these tools for research purposes only. All other enquiries should be sent to jbisynthesis@adelaide.edu.au.

Reviewer SILVIA PAVONE/PAOLA GOBBI Date__________25/09/2023_______________

Author Pisoni et al., Year 2007 Record Number 75

	Yes	No	Unclear	Not applicable
1. Were the criteria for inclusion in the sample clearly defined?	■	□	□	□
2. Were the study subjects and the setting described in detail?	□	■	□	□
3. Was the exposure measured in a valid and reliable way?	■	□	□	□
4. Were objective, standard criteria used for measurement of the condition?	■	□	□	□
5. Were confounding factors identified?	□	□	□	■
6. Were strategies to deal with confounding factors stated?	□	□	□	■
7. Were the outcomes measured in a valid and reliable way and expressed clearly? Has been used any reliable statistical methods to evaluate the phylogenetic results?	□	■	□	□
8. Was appropriate statistical analysis used?	■	□	□	□

Overall appraisal: Include ■ Exclude □ Seek further info □

Comments (Including reason for exclusion)

Criticality: missing data (time period), there is no correlation between accession numbers and subgenotypes

Ranking: good (66%)

© JBI, 2020. All rights reserved. JBI grants use of these tools for research purposes only. All other enquiries should be sent to jbisynthesis@adelaide.edu.au.

Reviewer SILVIA PAVONE/PAOLA GOBBI Date_________25/09/2023______________

Author Pisoni et al., Year 2010 Record Number 76

	Yes	No	Unclear	Not applicable
1. Were the criteria for inclusion in the sample clearly defined?	■	□	□	□
2. Were the study subjects and the setting described in detail?	□	■	□	□
3. Was the exposure measured in a valid and reliable way?	■	□	□	□
4. Were objective, standard criteria used for measurement of the condition?	■	□	□	□
5. Were confounding factors identified?	□	□	□	■
6. Were strategies to deal with confounding factors stated?	□	□	□	■
7. Were the outcomes measured in a valid and reliable way and expressed clearly? Has been used any reliable statistical methods to evaluate the phylogenetic results?	□	■	□	□
8. Was appropriate statistical analysis used?	■	□	□	□

Overall appraisal: Include ■ Exclude □ Seek further info □

Comments (Including reason for exclusion)

Criticality: missing data (time period), there is no correlation between accession numbers and subgenotypes

Ranking: good (66%)

© JBI, 2020. All rights reserved. JBI grants use of these tools for research purposes only. All other enquiries should be sent to jbisynthesis@adelaide.edu.au.

Reviewer SILVIA PAVONE/PAOLA GOBBI Date_________25/09/2023______________

Author Pisoni et al., Year 2007 Record Number 77

	Yes	No	Unclear	Not applicable
9. Were the criteria for inclusion in the sample clearly defined?	■	□	□	□
10. Were the study subjects and the setting described in detail?	□	■	□	□
11. Was the exposure measured in a valid and reliable way?	■	□	□	□
12. Were objective, standard criteria used for measurement of the condition?	■	□	□	□
13. Were confounding factors identified?	□	□	□	■
14. Were strategies to deal with confounding factors stated?	□	□	□	■
15. Were the outcomes measured in a valid and reliable way and expressed clearly? Has been used any reliable statistical methods to evaluate the phylogenetic results?	□	■	□	□
16. Was appropriate statistical analysis used?	■	□	□	□

Overall appraisal: Include ■ Exclude □ Seek further info □

Comments (Including reason for exclusion)

Criticality: missing data (time period), there is no correlation between accession numbers and subgenotypes

Ranking: good (66%)

© JBI, 2020. All rights reserved. JBI grants use of these tools for research purposes only. All other enquiries should be sent to jbisynthesis@adelaide.edu.au.

Reviewer SILVIA PAVONE/PAOLA GOBBI Date_________25/09/2023______________

Author Pisoni et al., Year 2005 Record Number 78

	Yes	No	Unclear	Not applicable
1. Were the criteria for inclusion in the sample clearly defined?	■	□	□	□
2. Were the study subjects and the setting described in detail?	■	□	□	□
3. Was the exposure measured in a valid and reliable way?	■	□	□	□
4. Were objective, standard criteria used for measurement of the condition?	■	□	□	□
5. Were confounding factors identified?	□	□	□	■
6. Were strategies to deal with confounding factors stated?	□	□	□	■
7. Were the outcomes measured in a valid and reliable way and expressed clearly? Has been used any reliable statistical methods to evaluate the phylogenetic results?	■	□	□	□
8. Was appropriate statistical analysis used?	■	□	□	□

Overall appraisal: Include ■ Exclude □ Seek further info □

Comments (Including reason for exclusion)

Ranking: very good (100%)

© JBI, 2020. All rights reserved. JBI grants use of these tools for research purposes only. All other enquiries should be sent to jbisynthesis@adelaide.edu.au.

Reviewer SILVIA PAVONE/PAOLA GOBBI Date__________25/09/2023_______________

Author Ramirez et al., Year 2011 Record Number 80

	Yes	No	Unclear	Not applicable
1. Were the criteria for inclusion in the sample clearly defined?	■	□	□	□
2. Were the study subjects and the setting described in detail?	□	■	□	□
3. Was the exposure measured in a valid and reliable way?	■	□	□	□
4. Were objective, standard criteria used for measurement of the condition?	■	□	□	□
5. Were confounding factors identified?	□	□	□	■
6. Were strategies to deal with confounding factors stated?	□	□	□	■
7. Were the outcomes measured in a valid and reliable way and expressed clearly? Has been used any reliable statistical methods to evaluate the phylogenetic results?	□	■	□	□
8. Was appropriate statistical analysis used?	□	□	■	□

Overall appraisal: Include ■ Exclude □ Seek further info □

Comments (Including reason for exclusion)

Criticality: missing data (time period, sequencing method), no details are given about phylogenetic analysis softwares

Ranking: unsatisfactory (58%)

© JBI, 2020. All rights reserved. JBI grants use of these tools for research purposes only. All other enquiries should be sent to jbisynthesis@adelaide.edu.au.

Reviewer SILVIA PAVONE/PAOLA GOBBI Date__________25/09/2023_______________

Author Ravazzolo et al., Year 2001 Record Number 81

	Yes	No	Unclear	Not applicable
1. Were the criteria for inclusion in the sample clearly defined?	■	□	□	□
2. Were the study subjects and the setting described in detail?	□	■	□	□
3. Was the exposure measured in a valid and reliable way?	■	□	□	□
4. Were objective, standard criteria used for measurement of the condition?	■	□	□	□
5. Were confounding factors identified?	□	□	□	■
6. Were strategies to deal with confounding factors stated?	□	□	□	■
7. Were the outcomes measured in a valid and reliable way and expressed clearly? Has been used any reliable statistical methods to evaluate the phylogenetic results?	■	□	□	□
8. Was appropriate statistical analysis used?	■	□	□	□

Overall appraisal: Include ■ Exclude □ Seek further info □

Comments (Including reason for exclusion)

Criticality: missing data (time period)

Ranking: very good (83%)

© JBI, 2020. All rights reserved. JBI grants use of these tools for research purposes only. All other enquiries should be sent to jbisynthesis@adelaide.edu.au.

Reviewer SILVIA PAVONE/PAOLA GOBBI Date_________25/09/2023______________

Author Reina et al., Year 2010 Record Number 82

	Yes	No	Unclear	Not applicable
1. Were the criteria for inclusion in the sample clearly defined?	■	□	□	□
2. Were the study subjects and the setting described in detail?	□	■	□	□
3. Was the exposure measured in a valid and reliable way?	■	□	□	□
4. Were objective, standard criteria used for measurement of the condition?	■	□	□	□
5. Were confounding factors identified?	□	□	□	■
6. Were strategies to deal with confounding factors stated?	□	□	□	■
7. Were the outcomes measured in a valid and reliable way and expressed clearly? Has been used any reliable statistical methods to evaluate the phylogenetic results?	■	□	□	□
8. Was appropriate statistical analysis used?	■	□	□	□

Overall appraisal: Include ■ Exclude □ Seek further info □

Comments (Including reason for exclusion)

Criticality: missing data (time period)

Ranking: very good (83%)

© JBI, 2020. All rights reserved. JBI grants use of these tools for research purposes only. All other enquiries should be sent to jbisynthesis@adelaide.edu.au.

Reviewer SILVIA PAVONE/PAOLA GOBBI Date__________25/09/2023_______________

Author Reina et al., Year 2009 Record Number 83

	Yes	No	Unclear	Not applicable
1. Were the criteria for inclusion in the sample clearly defined?	■	□	□	□
2. Were the study subjects and the setting described in detail?	□	■	□	□
3. Was the exposure measured in a valid and reliable way?	■	□	□	□
4. Were objective, standard criteria used for measurement of the condition?	■	□	□	□
5. Were confounding factors identified?	□	□	□	■
6. Were strategies to deal with confounding factors stated?	□	□	□	■
7. Were the outcomes measured in a valid and reliable way and expressed clearly? Has been used any reliable statistical methods to evaluate the phylogenetic results?	■	□	□	□
8. Was appropriate statistical analysis used?	■	□	□	□

Overall appraisal: Include ■ Exclude □ Seek further info □

Comments (Including reason for exclusion)

Criticality: missing data (time period)

Ranking: very good (83%)

© JBI, 2020. All rights reserved. JBI grants use of these tools for research purposes only. All other enquiries should be sent to jbisynthesis@adelaide.edu.au.

Reviewer SILVIA PAVONE/PAOLA GOBBI Date_________25/09/2023______________

Author Reina et al., Year 2009 Record Number 84

	Yes	No	Unclear	Not applicable
1. Were the criteria for inclusion in the sample clearly defined?	■	□	□	□
2. Were the study subjects and the setting described in detail?	□	■	□	□
3. Was the exposure measured in a valid and reliable way?	■	□	□	□
4. Were objective, standard criteria used for measurement of the condition?	■	□	□	□
5. Were confounding factors identified?	□	□	□	■
6. Were strategies to deal with confounding factors stated?	□	□	□	■
7. Were the outcomes measured in a valid and reliable way and expressed clearly? Has been used any reliable statistical methods to evaluate the phylogenetic results?	□	■	□	□
8. Was appropriate statistical analysis used?	■	□	□	□

Overall appraisal: Include ■ Exclude □ Seek further info □

Comments (Including reason for exclusion)

Criticality: missing data (time period, sequencing method), there is no correlation between accession numbers and subgenotypes

Ranking: good (66%)

© JBI, 2020. All rights reserved. JBI grants use of these tools for research purposes only. All other enquiries should be sent to jbisynthesis@adelaide.edu.au.

Reviewer SILVIA PAVONE/PAOLA GOBBI Date_________25/09/2023______________

Author Reina et al., Year 2006 Record Number 85

	Yes	No	Unclear	Not applicable
1. Were the criteria for inclusion in the sample clearly defined?	■	□	□	□
2. Were the study subjects and the setting described in detail?	□	■	□	□
3. Was the exposure measured in a valid and reliable way?	■	□	□	□
4. Were objective, standard criteria used for measurement of the condition?	■	□	□	□
5. Were confounding factors identified?	□	□	□	■
6. Were strategies to deal with confounding factors stated?	□	□	□	■
7. Were the outcomes measured in a valid and reliable way and expressed clearly? Has been used any reliable statistical methods to evaluate the phylogenetic results?	□	■	□	□
8. Was appropriate statistical analysis used?	■	□	□	□

Overall appraisal: Include ■ Exclude □ Seek further info □

Comments (Including reason for exclusion)

Criticality: missing data (time period), there is no correlation between accession numbers and subgenotypes

Ranking: good (66%)

© JBI, 2020. All rights reserved. JBI grants use of these tools for research purposes only. All other enquiries should be sent to jbisynthesis@adelaide.edu.au.

Reviewer SILVIA PAVONE/PAOLA GOBBI Date_________25/09/2023______________

Author Rolland et al., Year 2002 Record Number 86

	Yes	No	Unclear	Not applicable
1. Were the criteria for inclusion in the sample clearly defined?	■	□	□	□
2. Were the study subjects and the setting described in detail?	□	■	□	□
3. Was the exposure measured in a valid and reliable way?	■	□	□	□
4. Were objective, standard criteria used for measurement of the condition?	■	□	□	□
5. Were confounding factors identified?	□	□	□	■
6. Were strategies to deal with confounding factors stated?	□	□	□	■
7. Were the outcomes measured in a valid and reliable way and expressed clearly? Has been used any reliable statistical methods to evaluate the phylogenetic results?	□	■	□	□
8. Was appropriate statistical analysis used?	■	□	□	□

Overall appraisal: Include ■ Exclude □ Seek further info □

Comments (Including reason for exclusion)

Criticality: missing data (time period, sequencing method, accession numbers and genotypes-subgenotypes)

Ranking: good (66%)

© JBI, 2020. All rights reserved. JBI grants use of these tools for research purposes only. All other enquiries should be sent to jbisynthesis@adelaide.edu.au.

Reviewer SILVIA PAVONE/PAOLA GOBBI Date__________25/09/2023_______________

Author Santry et al., Year 2013 Record Number 87

	Yes	No	Unclear	Not applicable
1. Were the criteria for inclusion in the sample clearly defined?	■	□	□	□
2. Were the study subjects and the setting described in detail?	□	■	□	□
3. Was the exposure measured in a valid and reliable way?	■	□	□	□
4. Were objective, standard criteria used for measurement of the condition?	■	□	□	□
5. Were confounding factors identified?	□	□	□	■
6. Were strategies to deal with confounding factors stated?	□	□	□	■
7. Were the outcomes measured in a valid and reliable way and expressed clearly? Has been used any reliable statistical methods to evaluate the phylogenetic results?	□	■	□	□
8. Was appropriate statistical analysis used?	■	□	□	□

Overall appraisal: Include ■ Exclude □ Seek further info □

Comments (Including reason for exclusion)

Criticality: missing data (time period), there is no correlation between accession numbers and subgenotypes

Ranking: good (66%)

© JBI, 2020. All rights reserved. JBI grants use of these tools for research purposes only. All other enquiries should be sent to jbisynthesis@adelaide.edu.au.

Reviewer SILVIA PAVONE/PAOLA GOBBI Date_________25/09/2023______________

Author Schaer et al., Year 2022 Record Number 88

	Yes	No	Unclear	Not applicable
1. Were the criteria for inclusion in the sample clearly defined?	■	□	□	□
2. Were the study subjects and the setting described in detail?	■	□	□	□
3. Was the exposure measured in a valid and reliable way?	■	□	□	□
4. Were objective, standard criteria used for measurement of the condition?	■	□	□	□
5. Were confounding factors identified?	□	□	□	■
6. Were strategies to deal with confounding factors stated?	□	□	□	■
7. Were the outcomes measured in a valid and reliable way and expressed clearly? Has been used any reliable statistical methods to evaluate the phylogenetic results?	□	■	□	□
8. Was appropriate statistical analysis used?	□	■	□	□

Overall appraisal: Include ■ Exclude □ Seek further info □

Comments (Including reason for exclusion)

Criticality: there is no correlation between accession numbers and genotypes, short genomic length, low bootstrap values in phylogenetic tree

Ranking: good (66%)

© JBI, 2020. All rights reserved. JBI grants use of these tools for research purposes only. All other enquiries should be sent to jbisynthesis@adelaide.edu.au.

Reviewer SILVIA PAVONE/PAOLA GOBBI Date_________25/09/2023______________

Author Shah et al., Year 2004 Record Number 89

	Yes	No	Unclear	Not applicable
1. Were the criteria for inclusion in the sample clearly defined?	■	□	□	□
2. Were the study subjects and the setting described in detail?	■	□	□	□
3. Was the exposure measured in a valid and reliable way?	■	□	□	□
4. Were objective, standard criteria used for measurement of the condition?	■	□	□	□
5. Were confounding factors identified?	□	□	□	■
6. Were strategies to deal with confounding factors stated?	□	□	□	■
7. Were the outcomes measured in a valid and reliable way and expressed clearly? Has been used any reliable statistical methods to evaluate the phylogenetic results?	□	■	□	□
8. Was appropriate statistical analysis used?	■	□	□	□

Overall appraisal: Include ■ Exclude □ Seek further info □

Comments (Including reason for exclusion)

Criticality: there is no correlation between accession numbers and subgenotypes

Ranking: very good (83%)

© JBI, 2020. All rights reserved. JBI grants use of these tools for research purposes only. All other enquiries should be sent to jbisynthesis@adelaide.edu.au.

Reviewer SILVIA PAVONE/PAOLA GOBBI Date_________25/09/2023______________

Author Shah et al., Year 2004 Record Number 90

	Yes	No	Unclear	Not applicable
1. Were the criteria for inclusion in the sample clearly defined?	■	□	□	□
2. Were the study subjects and the setting described in detail?	□	■	□	□
3. Was the exposure measured in a valid and reliable way?	■	□	□	□
4. Were objective, standard criteria used for measurement of the condition?	■	□	□	□
5. Were confounding factors identified?	□	□	□	■
6. Were strategies to deal with confounding factors stated?	□	□	□	■
7. Were the outcomes measured in a valid and reliable way and expressed clearly? Has been used any reliable statistical methods to evaluate the phylogenetic results?	□	■	□	□
8. Was appropriate statistical analysis used?	■	□	□	□

Overall appraisal: Include ■ Exclude □ Seek further info □

Comments (Including reason for exclusion)

Criticality: missing data (time period), there is no correlation between accession numbers and subgenotypes

Ranking: good (66%)

© JBI, 2020. All rights reserved. JBI grants use of these tools for research purposes only. All other enquiries should be sent to jbisynthesis@adelaide.edu.au.

Reviewer SILVIA PAVONE/PAOLA GOBBI Date_________25/09/2023______________

Author Sider et al., Year 2013 Record Number 91

	Yes	No	Unclear	Not applicable
1. Were the criteria for inclusion in the sample clearly defined?	■	□	□	□
2. Were the study subjects and the setting described in detail?	□	■	□	□
3. Was the exposure measured in a valid and reliable way?	■	□	□	□
4. Were objective, standard criteria used for measurement of the condition?	■	□	□	□
5. Were confounding factors identified?	□	□	□	■
6. Were strategies to deal with confounding factors stated?	□	□	□	■
7. Were the outcomes measured in a valid and reliable way and expressed clearly? Has been used any reliable statistical methods to evaluate the phylogenetic results?	□	■	□	□
8. Was appropriate statistical analysis used?	■	□	□	□

Overall appraisal: Include ■ Exclude □ Seek further info □

Comments (Including reason for exclusion)

Criticality: missing data (time period, bootstrap values), there is no correlation between accession numbers and subgenotypes

Ranking: good (66%)

© JBI, 2020. All rights reserved. JBI grants use of these tools for research purposes only. All other enquiries should be sent to jbisynthesis@adelaide.edu.au.

Reviewer SILVIA PAVONE/PAOLA GOBBI Date__________26/09/2023_______________

Author Valas et al., Year 2000 Record Number 92

	Yes	No	Unclear	Not applicable
1. Were the criteria for inclusion in the sample clearly defined?	■	□	□	□
2. Were the study subjects and the setting described in detail?	□	■	□	□
3. Was the exposure measured in a valid and reliable way?	■	□	□	□
4. Were objective, standard criteria used for measurement of the condition?	■	□	□	□
5. Were confounding factors identified?	□	□	□	■
6. Were strategies to deal with confounding factors stated?	□	□	□	■
7. Were the outcomes measured in a valid and reliable way and expressed clearly? Has been used any reliable statistical methods to evaluate the phylogenetic results?	□	■	□	□
8. Was appropriate statistical analysis used?	■	□	□	□

Overall appraisal: Include ■ Exclude □ Seek further info □

Comments (Including reason for exclusion)

Criticality: missing data (time period, sequencing method)

Ranking: good (66%)

© JBI, 2020. All rights reserved. JBI grants use of these tools for research purposes only. All other enquiries should be sent to jbisynthesis@adelaide.edu.au.

Reviewer SILVIA PAVONE/PAOLA GOBBI Date_________26/09/2023______________

Author Valas et al., Year 1997 Record Number 93

	Yes	No	Unclear	Not applicable
1. Were the criteria for inclusion in the sample clearly defined?	■	□	□	□
2. Were the study subjects and the setting described in detail?	□	■	□	□
3. Was the exposure measured in a valid and reliable way?	■	□	□	□
4. Were objective, standard criteria used for measurement of the condition?	■	□	□	□
5. Were confounding factors identified?	□	□	□	■
6. Were strategies to deal with confounding factors stated?	□	□	□	■
7. Were the outcomes measured in a valid and reliable way and expressed clearly? Has been used any reliable statistical methods to evaluate the phylogenetic results?	■	□	□	□
8. Was appropriate statistical analysis used?	■	□	□	□

Overall appraisal: Include ■ Exclude □ Seek further info □

Comments (Including reason for exclusion)

Criticality: missing data (time period)

Ranking: very good (83%)

© JBI, 2020. All rights reserved. JBI grants use of these tools for research purposes only. All other enquiries should be sent to jbisynthesis@adelaide.edu.au.

Reviewer SILVIA PAVONE/PAOLA GOBBI Date________26/09/2023_____________

Author Workman et al., Year 2018 Record Number 94

	Yes	No	Unclear	Not applicable
1. Were the criteria for inclusion in the sample clearly defined?	■	□	□	□
2. Were the study subjects and the setting described in detail?	□	■	□	□
3. Was the exposure measured in a valid and reliable way?	■	□	□	□
4. Were objective, standard criteria used for measurement of the condition?	■	□	□	□
5. Were confounding factors identified?	□	□	□	■
6. Were strategies to deal with confounding factors stated?	□	□	□	■
7. Were the outcomes measured in a valid and reliable way and expressed clearly? Has been used any reliable statistical methods to evaluate the phylogenetic results?	■	□	□	□
8. Was appropriate statistical analysis used?	■	□	□	□

Overall appraisal: Include ■ Exclude □ Seek further info □

Comments (Including reason for exclusion)

Criticality: missing data (time period)

Ranking: very good (83%)

© JBI, 2020. All rights reserved. JBI grants use of these tools for research purposes only. All other enquiries should be sent to jbisynthesis@adelaide.edu.au.

Reviewer SILVIA PAVONE/PAOLA GOBBI Date__________26/09/2023_______________

Author Workman et al., Year 2017 Record Number 95

	Yes	No	Unclear	Not applicable
1. Were the criteria for inclusion in the sample clearly defined?	■	□	□	□
2. Were the study subjects and the setting described in detail?	□	■	□	□
3. Was the exposure measured in a valid and reliable way?	■	□	□	□
4. Were objective, standard criteria used for measurement of the condition?	■	□	□	□
5. Were confounding factors identified?	□	□	□	■
6. Were strategies to deal with confounding factors stated?	□	□	□	■
7. Were the outcomes measured in a valid and reliable way and expressed clearly? Has been used any reliable statistical methods to evaluate the phylogenetic results?	■	□	□	□
8. Was appropriate statistical analysis used?	■	□	□	□

Overall appraisal: Include ■ Exclude □ Seek further info □

Comments (Including reason for exclusion)

Criticality: missing data (time period)

Ranking: very good (83%)

© JBI, 2020. All rights reserved. JBI grants use of these tools for research purposes only. All other enquiries should be sent to jbisynthesis@adelaide.edu.au.

Reviewer SILVIA PAVONE/PAOLA GOBBI Date_________26/09/2023______________

Author Wu et al., Year 2022 Record Number 96

	Yes	No	Unclear	Not applicable
1. Were the criteria for inclusion in the sample clearly defined?	■	□	□	□
2. Were the study subjects and the setting described in detail?	■	□	□	□
3. Was the exposure measured in a valid and reliable way?	■	□	□	□
4. Were objective, standard criteria used for measurement of the condition?	■	□	□	□
5. Were confounding factors identified?	□	□	□	■
6. Were strategies to deal with confounding factors stated?	□	□	□	■
7. Were the outcomes measured in a valid and reliable way and expressed clearly? Has been used any reliable statistical methods to evaluate the phylogenetic results?	■	□	□	□
8. Was appropriate statistical analysis used?	■	□	□	□

Overall appraisal: Include ■ Exclude □ Seek further info □

Comments (Including reason for exclusion)

Ranking: very good (100%)

© JBI, 2020. All rights reserved. JBI grants use of these tools for research purposes only. All other enquiries should be sent to jbisynthesis@adelaide.edu.au.

Reviewer SILVIA PAVONE/PAOLA GOBBI Date_________26/09/2023______________

Author Zanoni et al., Year 1998 Record Number 97

	Yes	No	Unclear	Not applicable
1. Were the criteria for inclusion in the sample clearly defined?	■	□	□	□
2. Were the study subjects and the setting described in detail?	■	□	□	□
3. Was the exposure measured in a valid and reliable way?	□	□	□	■
4. Were objective, standard criteria used for measurement of the condition?	□	□	□	■
5. Were confounding factors identified?	□	□	□	■
6. Were strategies to deal with confounding factors stated?	□	□	□	■
7. Were the outcomes measured in a valid and reliable way and expressed clearly? Has been used any reliable statistical methods to evaluate the phylogenetic results?	■	□	□	□
8. Was appropriate statistical analysis used?	■	□	□	□

Overall appraisal: Include ■ Exclude □ Seek further info □

Comments (Including reason for exclusion)

Observations: the study is based on data downloaded online, not on data produced by the authors

Ranking: very good (100%)

© JBI, 2020. All rights reserved. JBI grants use of these tools for research purposes only. All other enquiries should be sent to jbisynthesis@adelaide.edu.au.

Reviewer SILVIA PAVONE/PAOLA GOBBI Date__________26/09/2023________________

Author Zhao et al., Year 2021 Record Number 98

	Yes	No	Unclear	Not applicable
1. Were the criteria for inclusion in the sample clearly defined?	■	□	□	□
2. Were the study subjects and the setting described in detail?	□	■	□	□
3. Was the exposure measured in a valid and reliable way?	■	□	□	□
4. Were objective, standard criteria used for measurement of the condition?	■	□	□	□
5. Were confounding factors identified?	□	□	□	■
6. Were strategies to deal with confounding factors stated?	□	□	□	■
7. Were the outcomes measured in a valid and reliable way and expressed clearly? Has been used any reliable statistical methods to evaluate the phylogenetic results?	■	□	□	□
8. Was appropriate statistical analysis used?	■	□	□	□

Overall appraisal: Include ■ Exclude □ Seek further info □

Comments (Including reason for exclusion)

Criticality: missing data (time period)

Ranking: very good (83%)

© JBI, 2020. All rights reserved. JBI grants use of these tools for research purposes only. All other enquiries should be sent to jbisynthesis@adelaide.edu.au.

Reviewer SILVIA PAVONE/PAOLA GOBBI Date_________19/02/2024_____________

Author Carrozza et al. Year 2023 Record Number 1/2024

	Yes	No	Unclear	Not applicable
1. Were the criteria for inclusion in the sample clearly defined?	■	□	□	□
2. Were the study subjects and the setting described in detail?	■	□	□	□
3. Was the exposure measured in a valid and reliable way?	□	□	□	■
4. Were objective, standard criteria used for measurement of the condition?	□	□	□	■
5. Were confounding factors identified?	□	□	□	■
6. Were strategies to deal with confounding factors stated?	□	□	□	■
7. Were the outcomes measured in a valid and reliable way and expressed clearly? Has been used any reliable statistical methods to evaluate the phylogenetic results?	■	□	□	□
8. Was appropriate statistical analysis used?	■	□	□	□

Overall appraisal: Include ■ Exclude □ Seek further info □

Comments (Including reason for exclusion):

Observations: the study is based on data downloaded online, not on data produced by the authors

Ranking: very good (100%)

© JBI, 2020. All rights reserved. JBI grants use of these tools for research purposes only. All other enquiries should be sent to jbisynthesis@adelaide.edu.au.

Reviewer SILVIA PAVONE/PAOLA GOBBI Date__________19/02/2024_______________

Author Kolbasova et al. Year 2023 Record Number 2/2024

	Yes	No	Unclear	Not applicable
1. Were the criteria for inclusion in the sample clearly defined?	■	□	□	□
2. Were the study subjects and the setting described in detail?	■	□	□	□
3. Was the exposure measured in a valid and reliable way?	■	□	□	□
4. Were objective, standard criteria used for measurement of the condition?	■	□	□	□
5. Were confounding factors identified?	□	□	□	■
6. Were strategies to deal with confounding factors stated?	□	□	□	■
7. Were the outcomes measured in a valid and reliable way and expressed clearly? Has been used any reliable statistical methods to evaluate the phylogenetic results?	□	■	□	□
8. Was appropriate statistical analysis used?	■	□	□	□

Overall appraisal: Include ■ Exclude □ Seek further info □

Comments (Including reason for exclusion):

Criticality: short sequence used for phylogenetic analysis, low bootstrap values in phylogenetic tree

Ranking: very good (83%)

© JBI, 2020. All rights reserved. JBI grants use of these tools for research purposes only. All other enquiries should be sent to jbisynthesis@adelaide.edu.au.

Reviewer SILVIA PAVONE/PAOLA GOBBI Date_________19/02/2024______________

Author Olech et al. Year 2023 Record Number 4/2024

	Yes	No	Unclear	Not applicable
1. Were the criteria for inclusion in the sample clearly defined?	■	□	□	□
2. Were the study subjects and the setting described in detail?	■	□	□	□
3. Was the exposure measured in a valid and reliable way?	■	□	□	□
4. Were objective, standard criteria used for measurement of the condition?	■	□	□	□
5. Were confounding factors identified?	□	□	□	■
6. Were strategies to deal with confounding factors stated?	□	□	□	■
7. Were the outcomes measured in a valid and reliable way and expressed clearly? Has been used any reliable statistical methods to evaluate the phylogenetic results?	■	□	□	□
8. Was appropriate statistical analysis used?	■	□	□	□

Overall appraisal: Include ■ Exclude □ Seek further info □

Comments (Including reason for exclusion):

Ranking: very good (100%)

© JBI, 2020. All rights reserved. JBI grants use of these tools for research purposes only. All other enquiries should be sent to jbisynthesis@adelaide.edu.au.

Reviewer SILVIA PAVONE/PAOLA GOBBI Date_________19/02/2024______________

Author Olech and Kuzmak Year 2023 Record Number 5/2024

	Yes	No	Unclear	Not applicable
1. Were the criteria for inclusion in the sample clearly defined?	☐	■	☐	☐
2. Were the study subjects and the setting described in detail?	■	☐	☐	☐
3. Was the exposure measured in a valid and reliable way?	■	☐	☐	☐
4. Were objective, standard criteria used for measurement of the condition?	■	☐	☐	☐
5. Were confounding factors identified?	☐	☐	☐	■
6. Were strategies to deal with confounding factors stated?	☐	☐	☐	■
7. Were the outcomes measured in a valid and reliable way and expressed clearly? Has been used any reliable statistical methods to evaluate the phylogenetic results?	☐	■	☐	☐
8. Was appropriate statistical analysis used?	■	☐	☐	☐

Overall appraisal: Include ■ Exclude ☐ Seek further info ☐

Comments (Including reason for exclusion):

Criticality: missing data (time period), there is no correlation between accession numbers and subgenotypes

Ranking: good (66%)

© JBI, 2020. All rights reserved. JBI grants use of these tools for research purposes only. All other enquiries should be sent to jbisynthesis@adelaide.edu.au.

Reviewer SILVIA PAVONE/PAOLA GOBBI Date_________19/02/2024______________

Author Sait and Ince Year 2023 Record Number 7/2024

	Yes	No	Unclear	Not applicable
1. Were the criteria for inclusion in the sample clearly defined?	■	□	□	□
2. Were the study subjects and the setting described in detail?	□	■	□	□
3. Was the exposure measured in a valid and reliable way?	■	□	□	□
4. Were objective, standard criteria used for measurement of the condition?	■	□	□	□
5. Were confounding factors identified?	□	□	□	■
6. Were strategies to deal with confounding factors stated?	□	□	□	■
7. Were the outcomes measured in a valid and reliable way and expressed clearly? Has been used any reliable statistical methods to evaluate the phylogenetic results?	□	■	□	□
8. Was appropriate statistical analysis used?	■	□	□	□

Overall appraisal: Include ■ Exclude □ Seek further info □

Comments (Including reason for exclusion):

Criticality: unsatisfactory medical history and signalment, low bootstrap values in phylogenetic tree

Ranking: good (66%)

© JBI, 2020. All rights reserved. JBI grants use of these tools for research purposes only. All other enquiries should be sent to jbisynthesis@adelaide.edu.au.

Reviewer SILVIA PAVONE/PAOLA GOBBI Date_________19/02/2024______________

Author Wang et al Year 2023 Record Number 8/2024

	Yes	No	Unclear	Not applicable
1. Were the criteria for inclusion in the sample clearly defined?	■	□	□	□
2. Were the study subjects and the setting described in detail?	■	□	□	□
3. Was the exposure measured in a valid and reliable way?	■	□	□	□
4. Were objective, standard criteria used for measurement of the condition?	■	□	□	□
5. Were confounding factors identified?	□	□	□	■
6. Were strategies to deal with confounding factors stated?	□	□	□	■
7. Were the outcomes measured in a valid and reliable way and expressed clearly? Has been used any reliable statistical methods to evaluate the phylogenetic results?	■	□	□	□
8. Was appropriate statistical analysis used?	■	□	□	□

Overall appraisal: Include ■ Exclude □ Seek further info □

Comments (Including reason for exclusion):

Ranking: very good (100%)

© JBI, 2020. All rights reserved. JBI grants use of these tools for research purposes only. All other enquiries should be sent to jbisynthesis@adelaide.edu.au.
