# Supplementary material for: Molecular Characterization of Small Ruminant Lentiviruses in Sheep and Goats: A Systematic Review
Source: Animals (Basel). 2024 Dec 8;14(23):3545. doi: 10.3390/ani14233545 (PMC11640545; doi:10.3390/ani14233545)
Supplement: Supplementary file 1 [file animals-14-03545-s001.zip › Table S4.pdf]

JBI CRITICAL APPRAISAL CHECKLIST FOR CASE REPORTS

Reviewer SILVIA PAVONE/PAOLA GOBBI Date________03/10/2023_____________

Author Adedeji et al. Year 2013 Record Number 2

	Yes	No	Unclear	Not applicable
1. Were animal's characteristics clearly described?	■	☐	☐	☐
2. Was the animal's history clearly described and presented as a timeline?	■	☐	☐	☐
3. Was the current clinical condition of the animal on presentation clearly described?	■	☐	☐	☐
4. Were diagnostic tests or assessment methods and the results clearly described?	☐	■	☐	☐
5. Was the intervention(s) or treatment procedure(s) clearly described?	☐	☐	☐	■
6. Was the post-intervention clinical condition clearly described?	☐	☐	☐	■
7. Were adverse events (harms) or unanticipated events identified and described?	☐	☐	☐	■
8. Does the case report provide takeaway lessons?	■	☐	☐	☐

Overall appraisal: Include ☐ Exclude ☐ Seek further info ☐

Comments (Including reason for exclusion)

Criticality: It is not clear the bp length of the region used in the phylogenetic tree

Ranking: good (80%)

Reviewer SILVIA PAVONE/PAOLA GOBBI Date_________03/10/2023______________

Author Cecco et al Year 2022 Record Number 13

	Yes	No	Unclear	Not applicable
9. Were animal's characteristics clearly described?	☐	■	☐	☐
10. Was the animal's history clearly described and presented as a timeline?	■	☐	☐	☐
11. Was the current clinical condition of the animal on presentation clearly described?	■	☐	☐	☐
12. Were diagnostic tests or assessment methods and the results clearly described?	☐	☐	☐	■
13. Was the intervention(s) or treatment procedure(s) clearly described?	☐	☐	☐	■
14. Was the post-intervention clinical condition clearly described?	☐	☐	☐	■
15. Were adverse events (harms) or unanticipated events identified and described?	☐	☐	☐	■
16. Does the case report provide takeaway lessons?	■	☐	☐	☐

Overall appraisal: Include ■ Exclude ☐ Seek further info ☐

Comments (Including reason for exclusion)

Criticality: Missing data about animal history

Ranking: good (80%)

© JBI, 2020. All rights reserved. JBI grants use of these tools for research purposes only. All other enquiries should be sent to jbisynthesis@adelaide.edu.au.

Reviewer SILVIA PAVONE/PAOLA GOBBI Date________19/02/2024_____________

Author Persson et al Year 2023 Record Number 5/2024

	Yes	No	Unclear	Not applicable
17. Were animal's characteristics clearly described?	■	□	□	□
18. Was the animal's history clearly described and presented as a timeline?	■	□	□	□
19. Was the current clinical condition of the animal on presentation clearly described?	■	□	□	□
20. Were diagnostic tests or assessment methods and the results clearly described?	□	□	□	■
21. Was the intervention(s) or treatment procedure(s) clearly described?	□	□	□	■
22. Was the post-intervention clinical condition clearly described?	□	□	□	■
23. Were adverse events (harms) or unanticipated events identified and described?	□	□	□	■
24. Does the case report provide takeaway lessons?	■	□	□	□

Overall appraisal: Include ■ Exclude □ Seek further info □

Comments (Including reason for exclusion)

Ranking: very good (100%)

© JBI, 2020. All rights reserved. JBI grants use of these tools for research purposes only. All other enquiries should be sent to jbisynthesis@adelaide.edu.au.
